# Origin and Evolution of Marsupial-specific Imprinting Clusters Through Lineage-specific Gene Duplications and Acquisition of Promoter Differential Methylation

**DOI:** 10.1093/molbev/msad022

**Published:** 2023-01-31

**Authors:** Wenqi Cao, Kory C Douglas, Paul B Samollow, John L VandeBerg, Xu Wang, Andrew G Clark

**Affiliations:** Department of Pathobiology, College of Veterinary Medicine, Auburn University, Auburn, AL, USA; Innovation, and Commerce, Alabama Agricultural Experiment Station, Auburn University Center for Advanced Science, Auburn, AL, USA; Department of Veterinary Integrative Biosciences, School of Veterinary Medicine and Biomedical Sciences, Texas A&M University, College Station, TX, USA; Department of Veterinary Integrative Biosciences, School of Veterinary Medicine and Biomedical Sciences, Texas A&M University, College Station, TX, USA; South Texas Diabetes and Obesity Institute and Department of Human Genetics, School of Medicine, The University of Texas Rio Grande Valley, Brownsville, TX, USA; Department of Pathobiology, College of Veterinary Medicine, Auburn University, Auburn, AL, USA; Innovation, and Commerce, Alabama Agricultural Experiment Station, Auburn University Center for Advanced Science, Auburn, AL, USA; Scott-Ritchey Research Center, College of Veterinary Medicine, Auburn University, Auburn, AL 36849, USA; HudsonAlpha Institute for Biotechnology, Huntsville, AL, USA; Department of Molecular Biology and Genetics, Cornell University, Ithaca, NY, USA

**Keywords:** *Monodelphis domestica*, opossum, genomic imprinting, fetal brain, placenta, DNA methylation

## Abstract

Genomic imprinting is a parent-of-origin-specific expression phenomenon that plays fundamental roles in many biological processes. In animals, imprinting is only observed in therian mammals, with ∼200 imprinted genes known in humans and mice. The imprinting pattern in marsupials has been minimally investigated by examining orthologs to known eutherian imprinted genes. To identify marsupial-specific imprinting in an unbiased way, we performed RNA-seq studies on samples of fetal brain and placenta from the reciprocal cross progeny of two laboratory opossum stocks. We inferred allele-specific expression for >3,000 expressed genes and discovered/validated 13 imprinted genes, including three previously known imprinted genes, *Igf2r*, *Peg10*, and *H19*. We estimate that marsupials imprint ∼60 autosomal genes, which is a much smaller set compared with eutherians. Among the nine novel imprinted genes, three noncoding RNAs have no known homologs in eutherian mammals, while the remaining genes have important functions in pluripotency, transcription regulation, nucleolar homeostasis, and neural differentiation. Methylation analyses at promoter CpG islands revealed differentially methylated regions in five of these marsupial-specific imprinted genes, suggesting that differential methylation is a common mechanism in the epigenetic regulation of marsupial imprinting. Clustering and co-regulation were observed at marsupial imprinting loci *Pou5f3*-*Npdc1* and *Nkrfl*-*Ipncr2*, but eutherian-type multi-gene imprinting clusters were not detected. Also differing from eutherian mammals, the brain and placenta imprinting profiles are remarkably similar in opossums, presumably due to the shared origin of these organs from the trophectoderm. Our results contribute to a fuller understanding of the origin, evolution, and mechanisms of genomic imprinting in therian mammals.

## Introduction

In diploid organisms, there are two sets of chromosomes, each individual set having been inherited from the mother or father, respectively. Approximately equal expression from both parental alleles is generally observed. However, a small number of genes show parent-of-origin-specific gene expression, in which only a single allele of either paternal origin or maternal origin is expressed. Such parent-of-origin-specific gene expression in diploid cells is regulated epigenetically and known as genomic imprinting (Barlow and Bartolomei 2014). In animals, genomic imprinting is found in therian mammals, that is eutherian and marsupial mammals, but has not been detected in prototherian mammals (Ferguson-Smith 2011).

In the 1980s, scientists found that the two sets of mammalian chromosomes can function differently, and both maternal and paternal sets are required for development, which suggested the existence of genomic imprinting for the first time (Barton et al. 1984; McGrath and Solter 1984; Surani et al. 1984; Cattanach and Kirk 1985). The first imprinted gene identified was *Igf2r* in mice (Barlow et al. 1991). It is a maternally expressed imprinted gene (MEG), which is associated with prenatal growth. More than 200 imprinted genes have now been identified in mice, and a slightly smaller number in humans (Tucci et al. 2019). Research in 2008 showed more than 80% of the 144 known imprinted genes known at that time were clustered into 16 genomic regions (Wan and Bartolomei 2008). An imprinting cluster can be composed of several protein-coding, maternally or paternally expressed imprinted genes (PEGs) and at least one lncRNA (Barlow and Bartolomei 2014). The imprinting status of the cluster can be controlled by differential methylation at imprinting control regions (ICRs), or other parent-of-origin epigenetic modifications (Edwards and Ferguson-Smith 2007; Barlow 2011).

Despite their small proportion among all mammalian genes (1–2%), imprinted genes are important players in many biologic processes, especially in embryonic and neonatal growth, development, and neurological function, and most of the imprinted genes are expressed in the placenta and brain (Tucci et al. 2019). Numerous studies have clearly described the significant functions of imprinted genes during prenatal development, including placentation and embryonic growth, which have been extensively reviewed (Cleaton et al. 2014). Neurological development is another fundamental process in which imprinted genes can cause postnatal pathologies, including neurological defects, altered brain function, and abnormal behavior, presenting in both young and adults (Reik 1989; Wilkinson et al. 2007). It is worth noting that many PEGs are growth promoters, while the first few maternally expressed imprinted genes discovered were found to repress the growth of the offspring, which can be explained by the parental conflict hypothesis (Moore and Haig 1991). Under the parental conflict hypothesis, paternal fitness is maximized by having his progeny gain more nutrients from the mother than the progeny of another father, while the mother's fitness is maximized by distributing her resources evenly among and within all her litters. Thus, conflict arises between paternal and maternal genomes due to opposing fitness consequences of the allocation of resources to progeny. This has been proposed as an evolutionary explanation for why the function of several identified imprinted genes is associated with fetal growth and postnatal nutritionally related behaviors (Curley et al. 2004; Haig 2004; Plagge et al. 2004; Schaller et al. 2010).

Genomic imprinting is widespread among placental mammals (eutherians), and a subset of eutherian imprinted genes has been examined and confirmed to be imprinted in a few marsupials (metatherians), but none of them have proven to be imprinted in the egg-laying monotremes (prototherians) (John and Surani 2000; Killian et al. 2000; Killian et al. 2001; Edwards et al. 2008; Renfree et al. 2008). Both eutherians and marsupials are viviparous, but they deliver relatively precocial and altricial young, respectively (Blackbum 1999). The evolutionary origin of genomic imprinting is prior to the marsupial–eutherian split, which coincides with the emergence of viviparity and the evolutionary invention of the placenta (Renfree et al. 2008). Marsupials, which diverged from eutherians about 160 million years ago (Luo et al. 2011), have a fully functional choriovitelline placenta, which is simpler and considered primitive compared with the complex placentas of eutherians (Tyndale-Biscoe and Renfree 1987). Differences in placentation and in utero development may explain the evolutionary origins of differential imprinting profiles and the regulation of imprinted gene expression between the two lineages (Renfree et al. 2009). Therefore, marsupials represent an alternative evolutionary path that can be examined in comparison to eutherians to better understand the origin, evolution, and regulation of genomic imprinting.

To date, there are 228 imprinted genes annotated in humans and 260 in mice, with 63 imprinted genes shared by both (Tucci et al. 2019). Twenty-nine of these have been investigated in marsupials, and of these 21 have marsupial orthologs (Pask 2012; Renfree et al. 2013). Among the 21 marsupial orthologs that have been examined, only 6 genes*, H19* (Smits et al. 2008), *Igf2* (O'Neill et al. 2000), *Igf2r* (Killian et al. 2000; Weidman et al. 2006), *Ins* (Ager et al. 2007; Das et al. 2012), *Mest/Peg1* (Suzuki et al. 2005), and *Peg10* (Suzuki et al. 2007) were confirmed to be imprinted. Previous studies focused on known eutherian imprinted genes, limiting the ability to discover marsupial-specific imprinted genes that are not imprinted in eutherians. The first ab initio search for marsupial imprinted genes was in *Monodelphis domestica* fibroblasts, in which one novel imprinted gene *Meis1*, and two monoallelically expressed genes were found through genome-wide ChIP-seq (Douglas et al. 2014). Due to the sporadic nature of existing studies, the marsupial genomic imprinting profile is far from complete. A systematic genome-wide and unbiased analysis of imprinted genes is needed to obtain more comprehensive conclusions about marsupial genomic imprinting.

DNA methylation and histone modifications are two major epigenetic regulatory mechanisms for eutherian imprinted genes. Differentially methylated regions (DMRs) are present in most eutherian imprinted gene clusters and are believed to cause differential allelic expression (Li et al. 1993). Deletion of a DMR leads to loss of imprinting for multiple genes in the same cluster (Barlow and Bartolomei 2014). Limited information about imprinting DMRs is available in marsupials: *Peg10* was shown to have a promoter DMR, and *H19* has a DMR upstream of the promoter in tammar wallabies (*Macropus eugenii*) (Suzuki et al. 2007; Smits et al. 2008). Another DMR was identified in the intronic region of *Igf2r* (Das et al. 2012). In addition, histone modifications also play an important role in genomic imprinting. In canonical genomic imprinting, different histone modifications are associated with the regulation of ICRs of imprinted genes (Delaval and Feil 2004). For example, ICRs of methylated unexpressed alleles display transcriptionally repressive modifications, including histone H3-lysine 9 trimethylation (H3K9me3), while marks of transcriptional activity such as H3-lysine 4 trimethylation (H3K4me3) and H3-lysine 9 acetylation (H3K9Ac), are present at the ICRs of the unmethylated expressed alleles (Fournier et al. 2002; Delaval and Feil 2004; Mikkelsen, Ku, et al. 2007). Recent work also demonstrated a DNA methylation-independent form of imprinting, a noncanonical form of imprinting, which is mediated by maternal H3K27me3 and showed distinct genomic characteristics and underlying mechanisms (Inoue et al. 2017). The previously mentioned genome-wide histone modification study of *M. domestica* fibroblasts (Douglas et al. 2014) revealed transcriptionally opposing histone modifications at the promoters of a novel imprinted gene and two monoallelically expressed genes, indicating that histone modification plays a conserved role in the genomic imprinting of marsupial animals as well.

The gray, short-tailed opossum, tammar wallaby, and Virginia opossum (*Didelphis virginiana*) are the most widely used marsupial species in laboratory research. Among them, *M. domestica* is the most well-developed model due to its high-quality genome assembly (Mikkelsen, Wakefield, et al. 2007), an extensive history of laboratory development and characterization, and the availability of well-characterized stocks and strains with diverse genetic backgrounds (Xiong et al. 2022), which enable crosses to track the parental origin of alleles for genomic imprinting research. To fill the knowledge gap in the expression profile and epigenetic regulation of marsupial imprinting, we performed transcriptome-wide RNA-seq analysis in fetal brain and extra-embryonic membranes (placental tissue) from reciprocal crosses of two *M. domestica* genetic stocks, LL1 and LL2. Thirteen candidate imprinted genes were identified, including 10 genes that were not previously known to be imprinted in any eutherian or marsupial species. This paper describes the first unbiased survey of genomic imprinting in marsupials, and sheds light on mechanisms of epigenetic regulation and the evolution of genomic imprinting in therian mammals.

## Materials and Methods

### Animals, Crosses, and Sample Collection Methods

The animal stocks, crosses, sample choices, and collection methods for this study have been described in detail in the Supplemental Methods section of Wang et al. 2014 (Wang et al. 2014). In brief, two random-bred stocks of *M. domestica*, LL1, and LL2, were selected to perform reciprocal crosses. The LL1 and LL2 stocks were derived over many years from two distinct groups of founder animals originally collected from different geographic regions in eastern Brazil as described by VandeBerg (Vandeberg and Williams-Blangero 2010). The LL1 stock was derived entirely from Population 1. LL2 was derived by admixture of Population 1 and Population 2 animals and comprises approximately 1:7 content of these two genetic backgrounds, respectively. Three LL1 animals (Females: A0578, A0580; Male: A0579) and three LL2 animals (Females: A0571, A0572; Male: A0573) were used for parental crosses and reciprocal crosses (supplementary Table S1, Supplementary Material online).

To obtain tissue samples from fetuses at known developmental stages, pair matings were performed for the parental and F_1_ crosses, with exact time of copulation determined from video recordings made for each mating. Eight embryonic day 13 (E13) fetal individuals and their respective placentas (extra-embryonic membranes, EEM) were collected from euthanized mothers and used for tissue dissections and RNA extractions: two female fetuses from each reciprocal cross (LL1×LL2 and LL2×LL1); one male and one female fetus from each parental cross (LL1×LL1 and LL2×LL2). Limbs and torsos of each fetus were also collected and used for genomic DNA extractions and tissue archiving (supplementary Table S1, Supplementary Material online).

### RNA-seq Library Construction and Data Analysis

A total of 16 RNA-seq libraries were made using the Illumina TruSeq RNA Sample Prep Kit (Illumina Inc., CA, USA) with 1–3 μg total RNA input. They were multiplexed and sequenced on an Illumina HiSeq 2000 platform (Illumina Inc.). A total of 1.5 billion 51-bp short reads were generated from the 16 samples. Detailed alignment and expression level quantification methods can be found in Wang et al. 2014 (Wang et al. 2014). In the eight brain samples, 13,092 Ensembl gene models were detected at FPKM ≥ 1 (Fragments Per Kilobase of transcript per Million mapped reads), and 11,890 genes were covered in the eight placental samples. The RNA-seq data were previously deposited in the Gene Expression Omnibus (GEO) database with accession number GSE45211.

### *De Novo* Transcript Assembly for Eutherian Imprinted Genes in the *igf2*-*H19* Imprinting Cluster

The *Igf2*-*H19* imprinting gene cluster is missing from the laboratory opossum reference genome monDom5 (Mikkelsen, Wakefield, et al. 2007). To include the eutherian imprinted genes of this cluster in our analysis, we performed de novo transcript contig assembly from quality filtered and trimmed reads combined using Trinity v2.4.0 (Haas et al. 2013). Transcript contigs of *Peg10*, *Igf2*, *H19*, *Ins2*, and *Cdkn1c* genes were included in the reference genome for SNP discovery and allele-specific gene expression analyses.

### Quantification of Parent-of-origin Allelic Expression and Detection of Imprinted Genes

Reads mapped to multiple places in the genome were filtered out in the BAM files. *De novo* SNP calling was performed on a combined BAM file of 16 transcriptomes using SAMtools (Li et al. 2009). More than 168,600 candidate autosomal exonic SNP positions were identified with a cut-off of 40× depth or higher. The reference and alternative allele counts were summarized for each SNP position in each individual transcriptome, and high-quality SNP positions with a total read depth of ≥8 in all individual transcriptomes were included for subsequent allele-specific gene expression analyses (Supplementary Data S1 and S2, Supplementary Material online). Informative SNPs were defined positions with both reference and alternative allele calls in samples from both parental LL1 and LL2 stocks, at least an 8× depth, and covered in at least three of the four F1 samples. SNPs located in repetitive regions or near exon–intron boundaries were excluded from further analyses. To estimate relative allelic expression ratios, the number of reference allele-containing reads was divided by the total coverage at each identified high-quality SNP position (Wang et al. 2008, 2011, 2013). Allele-specific expression ratios were computed for a total of ∼60,000 SNPs.

### SNP Genotype Confirmation by Sanger Sequencing

To confirm the parental origin of alleles at heterozygous SNP sites, Sanger sequencing was performed to validate SNP genotypes called from the RNA-seq data, using parental DNA samples extracted from liver and F_1_ DNA samples extracted from fetal limbs. PCR and sequencing primers were designed using Primer3 software. ABI TaqGold polymerase (Applied Biosystems, MA, USA) was used for PCR amplification followed by gel purification to remove free nucleotide and non-specific PCR products. PCR amplicons were sequenced at Beckman Coulter Genomics (Danvers, MA, USA), and sequences were analyzed by Sequencher 4.10. Detailed experimental protocols were described in (Douglas 2013). Candidate imprinted genes verified to have informative heterozygous SNPs in at least one of the two reciprocal F1 crosses with trackable parent-of-origin-specific alleles (supplementary Table S2-S3, Supplementary Material online) were included in the analyses of their imprinting status (supplementary Table S4-S5, Supplementary Material online).

### Mapping Candidate Imprinted Genes From the Unplaced Scaffold to the X Chromosome

DNA Fluorescence *In Situ* Hybridization (DNA FISH) was used to map candidate imprinted genes on chrUn to the X chromosome. Bacterial artificial chromosome (BAC) clones that contain *Smc6* (VMRC16:415P26) and an X-linked marker gene (VMRC18:608C5) were used as probes. Detailed protocols can be found in Douglas (2013).

### Validation of Imprinted Gene Expression by Allele-specific Pyrosequencing

Allele-specific pyrosequencing was performed to validate the imprinting status of eight novel imprinted genes and one previously known imprinted gene, *Igf2r,* at informative SNP positions confirmed by Sanger sequencing. Pyrosequencing primers were designed using PyroMark Assay Design Software Version 2.0.1.15 (Qiagen, CA, USA) (supplementary Table S7, Supplementary Material online). Ampli-Taq Gold polymerase (Life Technologies, CA, USA) was used in PCR amplification. PCR products were prepared using PyroMark Gold Reagents (Qiagen, CA, USA). PSQ 96MA Pyrosequencer and PyroMark Q48 instruments (Qiagen, CA, USA) were used to run the samples with the Allele Quantification method. A minimum of two technical replicates was included for each assay.

### Promoter CpG Methylation Analyses by Bisulfite Treatment, Cloning, and Sanger Sequencing

*Npdc1* and *Smc6l* promoter CpG island (CGI) methylation analyses were performed by bisulfite sequencing using the cloning and Sanger sequencing approach. A total of 2 μg of genomic DNA was treated with sodium bisulfite using the EpiTech Bisulfite Kit (Qiagen, MD, USA). PCR primers were designed by Methyl Primer Express software v1.0 (Applied Biosystems, CA; supplementary Table S8, Supplementary Material online). The amplicons were cloned using Topo TA Cloning Kit (Life Technologies, CA, USA). Transformed colonies were selected and sequenced using M13 forward primer at Beckman Coulter Genomics (Danvers, MA, USA). The sequences were then analyzed using Sequencher 4.10.

### Promoter CpG Methylation Analyses Using PyroMark Assays

DNA methylation percentages at promoter CpG sites were quantified using the PyroMark assay. 500 ng of DNA was used for bisulfite conversion with the Qiagen EpiTect Bisulfite Kit (Qiagen, CA, USA). PyroMark PCR primers were designed for CGIs with the PyroMark Assay Design Software Version 2.0.1.15 (supplementary Table S8, Supplementary Material online). PyroMark Assays were performed on PSQ 96MA Pyrosequencer (Qiagen, CA, USA). The pyrograms and DNA methylation percentages were generated and determined by Qiagen PyroMark CpG software.

### Phylogenetic and Gene Neighborhood Analysis for Novel Imprinted Genes Identified in Opossum

Phylogenetic trees for novel imprinted genes *Pou5f3*, *Npdc1*, and *Nkrfl1/2*, as well as their paralogs and neighboring genes (*Pou5f1, Tcf19*, and *Nkrf*), were constructed using protein sequence alignments from 26 vertebrates that have genome assemblies (supplementary Table S9, Supplementary Material online). Protein sequences of selected genes in these species were extracted from GenBank and Ensembl databases (accession numbers listed in supplementary Tables S10-S11, Supplementary Material online) or through manual annotation (Supplementary Data S3, Supplementary Material online). Sequence alignments were performed using Mafft (version 7.475), with parameters L-INS-i (Katoh and Standley 2013). The best fitting model for protein evolution was determined by ProtTest (version 3.4.2) (Darriba et al. 2011) to construct phylogenetic trees using the maximum likelihood (ML) method in RAxML (version 8.2.12) based on the JTT model with 1,000 bootstrap replicates (Stamatakis 2014). The phylogenetic trees were visualized and edited in FigTree (version 1.4.4), with re-rooting and rotations. Syntenic relationships and conservation of gene order of the imprinted genes and adjacent genes were determined by examination of corresponding genome assemblies in the UCSC genome browser. Manual annotations based on tBLASTn were performed for misannotated genes, with a cut-off of 1 × 10^−10^ for opossum *Nkrfl1/2* genes, as well as Axolotl *Npdc1* and *Nkrf* genes.

## Results

### Quantification of Allele-specific Expression in *M. domestica* Fetal Brain and Placenta by Transcriptome-wide RNA-seq

Illumina RNA-seq was performed on *M. domestica* E13 fetal brain and placenta samples to determine the allele-specific gene expression ratios in reciprocal F1 hybrid crosses of LL1 and LL2 strains (see Materials and Methods and supplementary Table S1, Supplementary Material online). More than 80% of reads were uniquely mapped to the laboratory opossum reference genome (MonDom5). Approximately 57,000 high-quality SNPs were called and only informative SNPs were used to estimate allele-specific expression ratios (Supplementary Data S1 and S2, Supplementary Material online). These SNPs reside in 3,745 genes in the fetal brain and 3,400 genes in the placenta, with 3,025 overlapped genes, which account for at least 20% of the expressed genes in the fetal brain and placenta transcriptome (*N* = 13,210 with average RPKM > 1.0).

### Identification of Marsupial Imprinted Genes in Opossum Fetal Brain and Placenta

Candidate imprinted genes were detected in both tissues using an allelic imbalance cut-off of *p*1 > 0.65 and *p*2 < 0.35, where *p*1 = LL1 allelic expression ratio in LL1×LL2 cross and *p*2 = LL1 allelic expression ratio in LL2×LL1 cross, for MEG candidates (Wang and Clark 2014). A cut-off of *p*1 < 0.35 and *p*2 > 0.65 was used to identify PEG candidates. To exclude the possibility of strong *cis*-eQTL effects, we required a “flipped” allelic expression pattern in the reciprocal crosses to ensure true parent-of-origin effects (Wang and Clark 2014). As a first pass, we examined seven imprinted genes previously reported in marsupial species. Among them, *Igf2*, *Ins2*, and *Meis1* lacked informative SNP positions between LL1 and LL2 in our data to infer allelic expression. *Mest* displayed biallelic expression patterns in reciprocal F1s, suggesting that it is not imprinted in the fetal brain and placenta in laboratory opossum. *H19* expression was 100% from the maternal allele (supported by one informative SNP and one indel position between LL1 and LL2). *Igf2r* also showed preferential maternal expression (>90% from maternal allele; supplementary fig. S1, Supplementary Material online). Five informative SNPs were called in *Peg10* transcript, and all of them indicated monoallelic expression from the paternal allele. The identification of these three previously known imprinted genes in marsupials with correct imprinting direction serves as a proof of principle for the transcriptome-wide detection of imprinted genes in laboratory opossum.

### A Total of 9 PEG and 10 MEG Candidates Were Identified in the Fetal Brain and Placenta

**Fig. 1.**
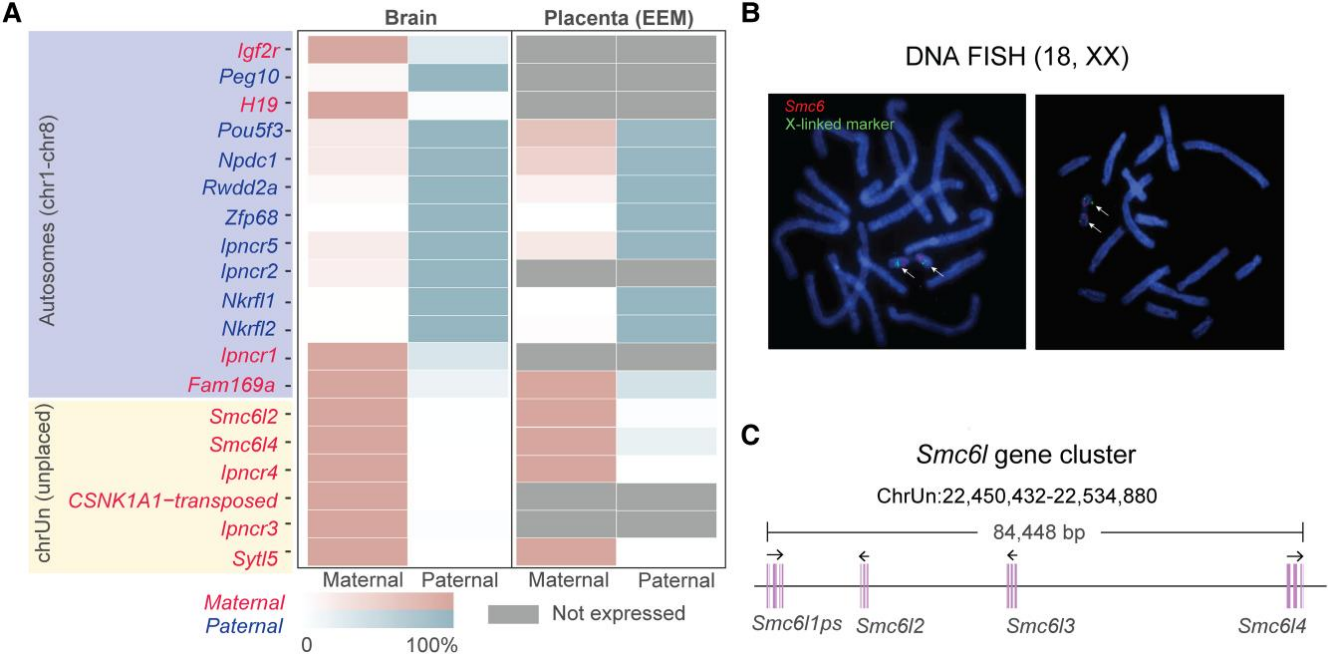
Genome location and parent-of-origin-specific expression profiles of 19 candidate autosomal imprinted genes identified in *Monodelphis domestica*. (*A*) Maternal and paternal allelic expression profiles of 19 candidate imprinted genes identified in *M. domestica* fetal brain and placenta (excluding genes on the X chromosome). Maternally and paternally expressed imprinted genes and the degree of parental bias in alelle-specific gene expression are labeled according to the legends. Undetectable genes in the corresponding tissue are shown in gray. The candidate imprinted genes on unplaced scaffolds (chrUn) were X-linked and subjected to imprinted X inactivation, with preferential expression from the maternal X chromosome (see B and C). (*B*) DNA FISH (Fluorescence *In Situ* Hybridization) using *Smc6l* BAC clone VMRC16:415P26 (red) and an X-linked BAC clone VMRC18:608C5 (green). The X chromosomes are labeled with arrows. (*C*) The schematic gene model plot of four *Smc6l* genes (*Smc6l1ps, Smc6l2, Smc6l3, and Smc6l4*) on *M. domestica* unplaced scaffold chrUn.

According to the imprinting candidate cut-off chosen, a total of 22 candidate imprinted genes were detected in the opossum fetal brain and placenta transcriptomes (supplementary Data S1–S2 and supplementary Tables S2–S3, Supplementary Material online). To confirm that SNPs used for allelic expression analysis are informative, we performed Sanger sequencing using genomic DNA from parental and F1 embryo samples (supplementary Table S4-S5, Supplementary Material online). Three “flipped” patterns in candidates, *Matn2*, *Prkaa2,* and *Parp4*, were the result of uninformative homozygous SNPs in reciprocal F_1_s (supplementary fig. S2, Supplementary Material online) and were excluded from the candidate list, leaving 19 candidates for further validation (fig. 1*A*). There were 9 PEG candidates and 10 MEG candidates in the fetal brain (fig. 1*A*), including 14 protein-coding genes and 5 *i*m*p*rinted *n*on*c*oding *R*NAs (*Ipncr1-5*). In the placenta sample, 12 imprinted candidates were identified in the RNA-seq data, and all of them were also imprinted in the fetal brain (fig. 1*A*).

### Validation of Autosomal Imprinting in *M. domestica*

**Fig. 2.**
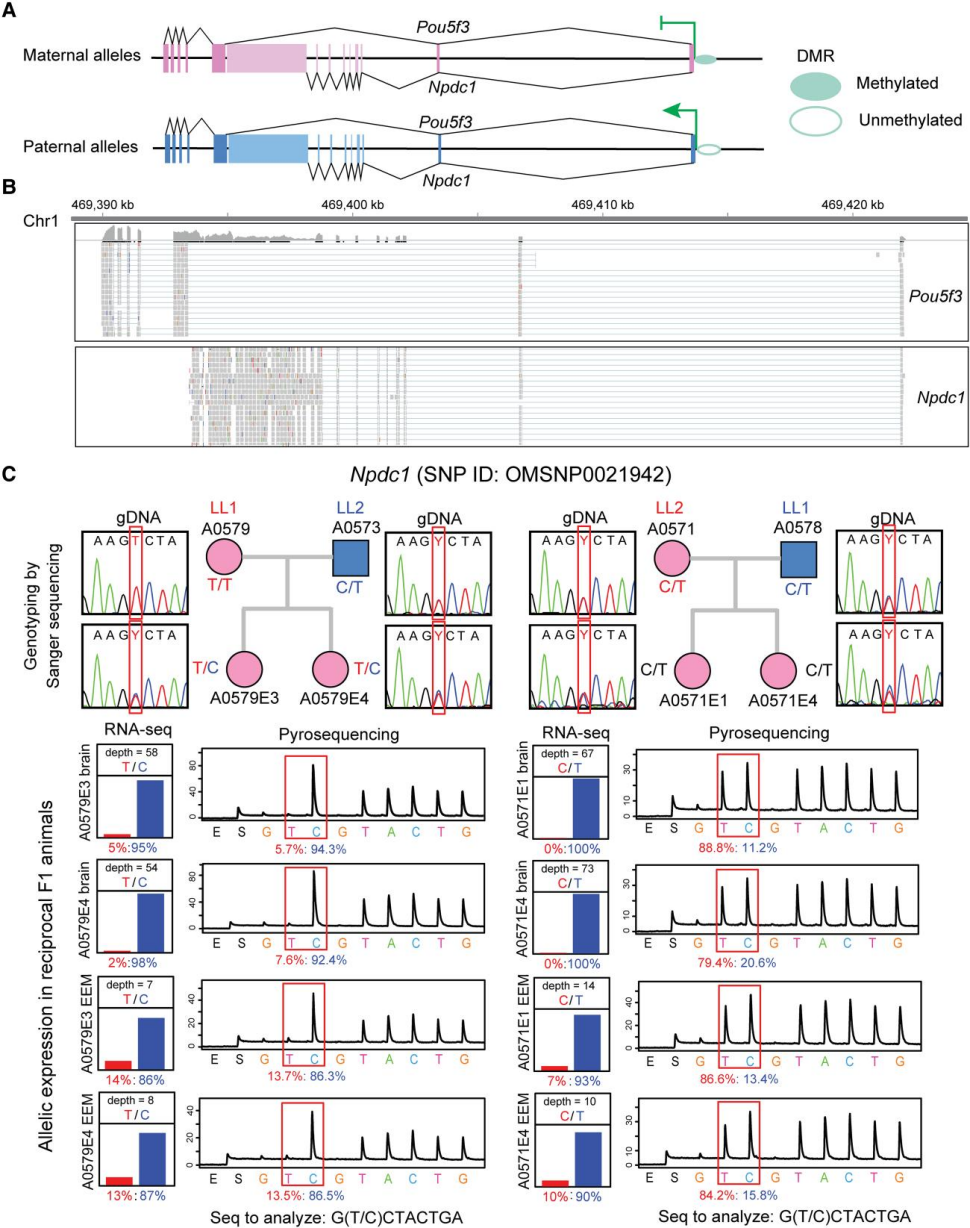
Gene structure and parent-of-origin-specific expression ratios at a novel imprinting cluster on chromosome 1 in *Monodelphis domestica* fetal brain and placenta. (*A*) *Npdc*1 and *Pou5f3* gene models inferred from RNA-seq data. Exons are represented by rectangle boxes, and DMRs are drawn as open (unmethylated) and filled (methylated) oval shapes. (*B*) RNA-seq read alignments in *Npdc1* and *Pou5f3* gene regions demonstrated by tracks from Integrative Genomics Viewer (IGV). (*C*) *Top*: *Npdc1* SNP genotypes in parental and F1 animals determined by Sanger sequencing in LL1 (dam) × LL2 (sire) and reciprocal LL2 × LL1 crosses. *Bottom*: *Npdc1* differential allelic expression profile estimated from RNA-seq (*left*) and validated by allele-specific pyrosequencing (*right*) in the two reciprocal crosses. The maternal allelic percentages are shown in red and paternal percentages are shown in blue.

**Fig. 3.**
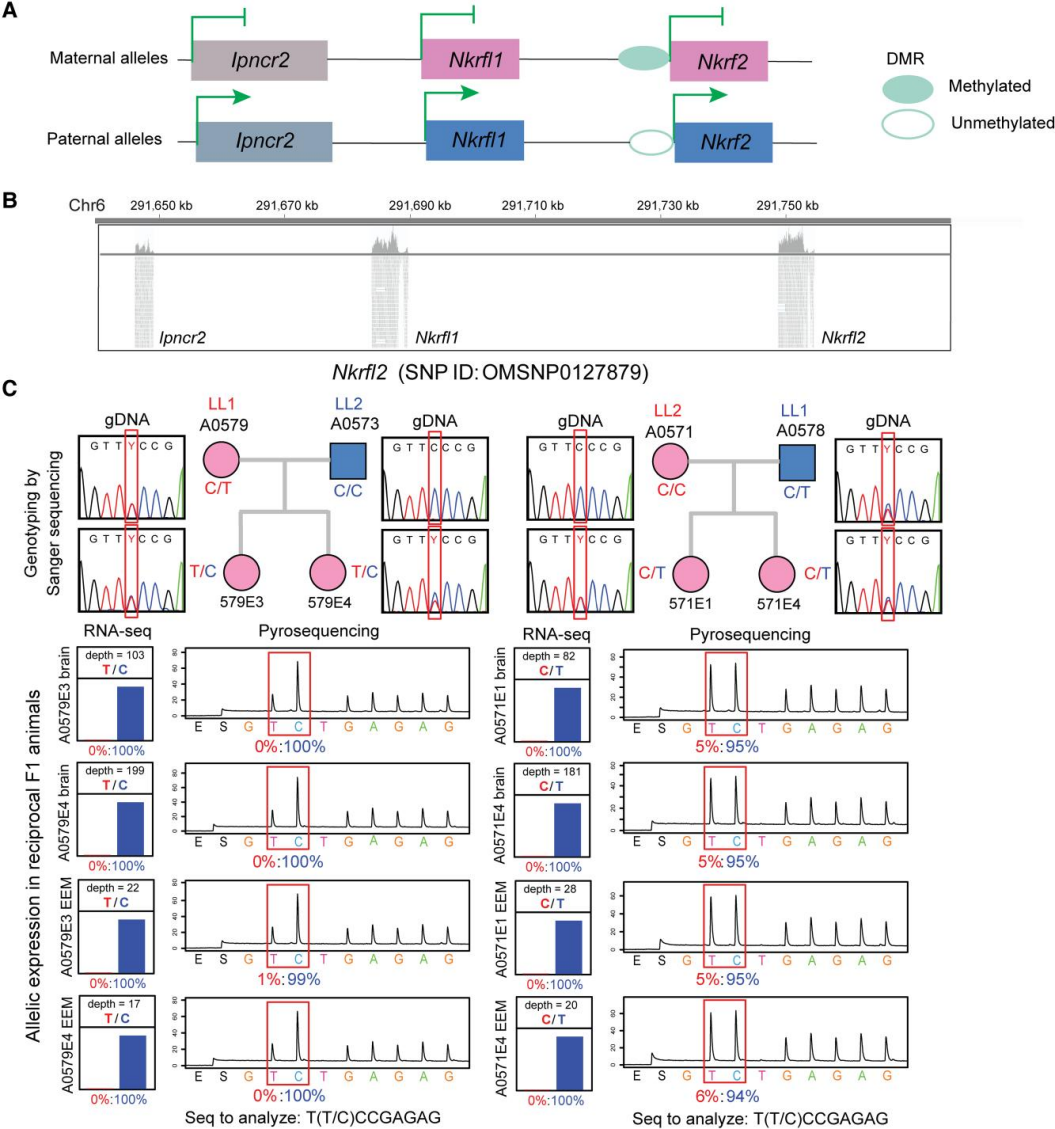
Gene structure and parent-of-origin allelic expression ratios at a novel imprinting cluster on chromosome 6 in *Monodelphis domestica* fetal brain and placenta. (*A*) *Nkrfl1*, *Nkrfl2*, and *Ipncr*2 gene models inferred from RNA-seq data. Exons are represented by rectangle boxes, and DMRs are drawn as open (unmethylated) and filled (methylated) oval shapes. (*B*) RNA-seq read alignments in *Nkrfl1*, *Nkrf2*, and the noncoding RNA gene *Ipncr*2 demonstrated by tracks from Integrative Genomics Viewer (IGV). (*C*) *Top*: *Nkrfl2* SNP genotypes in parental and F1 animals determined by Sanger sequencing in LL1 (dam) × LL2 (sire) and reciprocal LL2 × LL1 crosses. *Bottom*: *Nkrfl2* differential allelic expression profile estimated from RNA-seq (*left*) and validated by allele-specific pyrosequencing (*right*) in the two reciprocal crosses. The maternal allelic percentages are shown in red and paternal percentages are shown in blue.

Among the novel PEG/MEG candidates we discovered, eight are paternally expressed, and two are maternally expressed (Table 1). Maternal expression of the noncoding RNA *Ipncr1* was validated by allele-specific pyrosequencing in fetal brain (supplementary fig. S5, Supplementary Material online). The novel PEG candidates were validated by allele-specific pyrosequencing (figs. 2 and 3, supplementary S3–S4, and S6–S8, Supplementary Material online) and confirmed to be imprinted genes in *M. domestica*. Pyrosequencing was not performed for the MEG candidate *Fam169a* due to difficulties in primer design, but it has strong support from multiple SNPs in our RNA-seq data (supplementary fig. S9, Supplementary Material online).

**Table 1:** . Novel Imprinted Genes Identified in *Monodelphis domestica* Fetal Brain and Placenta (EEM).

Gene Name	Chr	Number of informative SNPs	Informative SNP positions	Tissue of imprinting	Expressed allele and allelic percentages			
					Fetal brain		EEM	
*Ipncr1*	Chr1	39	432,003,410	Fetal brain	Maternal	95.5%	Not Detectable	NA
			432,000,146					
			432,000,490					
*Pou5f3*	Chr1	2	469,394,133	Fetal brain	Paternal	97.8%	Paternal	71.6%
			469,390,121					
*Npdc1*	Chr1	7	469,395,728	Fetal brain and EEM	Paternal	97.7%	Paternal	90.0%
			469,398,410					
			469,398,225					
			469,397,930					
			469,398,122					
			469,397,875					
			469,397,620					
*Rwdd2a*	Chr2	3	338,819,188	Fetal brain and EEM	Paternal	99.4%	Paternal	98.6%
			338,819,003					
			338,819,189					
*Zfp68*	Chr2	1	522,422,185	Fetal brain and EEM	Paternal	100.0%	Paternal	100.0%
*Ipncr5*	Chr3	3	509,558,241	Fetal brain and EEM	Paternal	98.1%	Paternal	97.6%
			509,558,302					
			509,558,418					
*Ipncr2*	Chr6	4	291,644,506	Fetal brain	Paternal	98.6%	Not Detectable	NA
			291,644,516					
			291,647,867					
			291,648,088					
*Nkrfl1*	Chr6	3	291,686,863	Fetal brain and EEM	Paternal	99.9%	Paternal	100.0%
			291,686,866					
			291,689,830					
*Nkrfl2*	Chr6	3	291,750,260	Fetal brain and EEM	Paternal	99.9%	Paternal	99.7%
			291,751,561					
			291,751,098					
*Fam169a*	Chr3	2	49,751,281	Fetal brain and EEM	Maternal	98.6%	Maternal	94.2%
			49,787,572					

### Six MEGs on Unplaced Scaffolds are X-linked and Subject to Imprinted X Inactivation

Six MEG candidates were found to be located on chrUn (unplaced scaffold), and they all displayed 100% maternal expression (fig. 1*A*). Since the preponderance of genes on the opossum X chromosome are subject to imprinted X inactivation with 100% maternal expression (Wang et al. 2014), we suspected that these six MEG candidates might be X-linked. To test this hypothesis, we performed DNA FISH using *Smc6l*-containing BAC clones and verified X-linked BAC clones (see Materials and Methods). Our results clearly demonstrated that *Smc6l* colocalized with X-linked markers on the opossum X chromosome, confirming the X-linkage of the imprinted candidates on chrUn scaffold (fig. 1*B*). Further characterization of the *Smc6l* locus revealed an 84,448 bp gene cluster consisting of three protein-coding genes (*Smc6l2*, *Smc6l3*, *Smc6l4*) and one pseudogene *Smc6l1ps* (fig. 1*C*). *Smc6l2* and *Smc6l4* had informative SNPs showing 100% maternal expression (supplementary Table S6, Supplementary Material online). The imprinted noncoding transcript *Ipncr4* is on the same contig as the *Smc6l* cluster, so is also assumed to be X-linked. The remaining chrUn genes, *Ipncr3*, CSNK1A1-transposed, and *Sytl5*, are also potentially X-linked, but they were not included in our subsequent analyses of autosomal imprinting.

### The Discovery and Verification of two Novel Marsupial Imprinting Clusters on Chromosomes 1 and 6 in *M. domestica*

In addition to the six X-linked imprinted genes, 12 autosomal candidate imprinted genes were detected by our examination: three genes already known to be imprinted in marsupials (*Igf2r, H19,* and *Peg10*) and nine novel MEG/PEG candidates. To confirm the robustness of our conclusions regarding the imprinting status of the candidates, we used allele-specific pyrosequencing as an independent approach to validate parent-of-origin allelic expression skewing for a subset of the candidates: *Igf2r* and eight of the novel candidates (see Materials and Methods and supplementary Table S7, Supplementary Material online). The known imprinted gene *Igf2r* was validated as a MEG with >90% maternal expression. The two novel PEG candidates, *Pou5f3* and *Npdc1*, were clustered together on chromosome 1, transcribing in the same direction from the same start site and sharing their first two exons (fig. 2*A*). Both genes were highly expressed in the fetal brain and placenta (fig. 2*B*). Genotyping by Sanger sequencing confirmed heterozygous positions in F_1_ samples (fig. 2*C*), and allele-specific pyrosequencing validated the preferential paternal expression for *Npdc1* (>85% paternal expression; fig. 2*C*) and *Pou5f3* (supplementary fig. S3, Supplementary Material online). *Pou5f3* expression in fetal brain was nearly monoallelic, with more than 90% paternal allele expression (supplementary fig. S3, Supplementary Material online). However, there was a relaxation of imprinting in placenta, with ∼60% paternal expression in the LL1 × LL2 cross and ∼80% paternal expression in LL2 × LL1 (supplementary fig. S3, Supplementary Material online). *Pou5f3* and *Npdc1* have not been reported to be imprinted in any other vertebrates, thus these two genes form a novel and marsupial-specific imprinting cluster.

Another imprinted cluster we identified is located on chromosome 6, containing three single-exon genes, *Ipncr2*, *Nkrfl1*, and *Nkrfl2* (fig. 3*A*). *Nkrfl* genes are retrotransposed tandem duplications derived from X-linked *Nkrf* (Ensembl gene ID: ENSMODG00000049547). *Nkrfl1 and Nkrf2* were moderately expressed in the fetal brain with similar expression level in the RNA-seq data (fig. 3*B*), and monoallelic paternal expression was validated by allele-specific pyrosequencing (fig. 3*C*). *Ipncr2* is an uncharacterized noncoding RNA gene (UCSC N-SCAN gene symbol: chr6.6.536), located approximately 40 kb upstream of *Nkrfl1* (fig. 3*A*). No other gene models were found in the genomic region between *Ipncr2* and *Nkrfl1* in the reference genome annotation, and no transcribed sequences matching this region were detected in our RNA-seq data (fig. 3*B*). *Ipncr2* was confirmed to be imprinted in the fetal brain, while biallelic expression was observed in the placenta at a much lower expression level (supplementary fig. S4, Supplementary Material online).

### Differential Promoter CpG Methylation is Associated With Parental-specific Expression for Marsupial Imprinted Genes that are Protein-coding

**Fig. 4.**
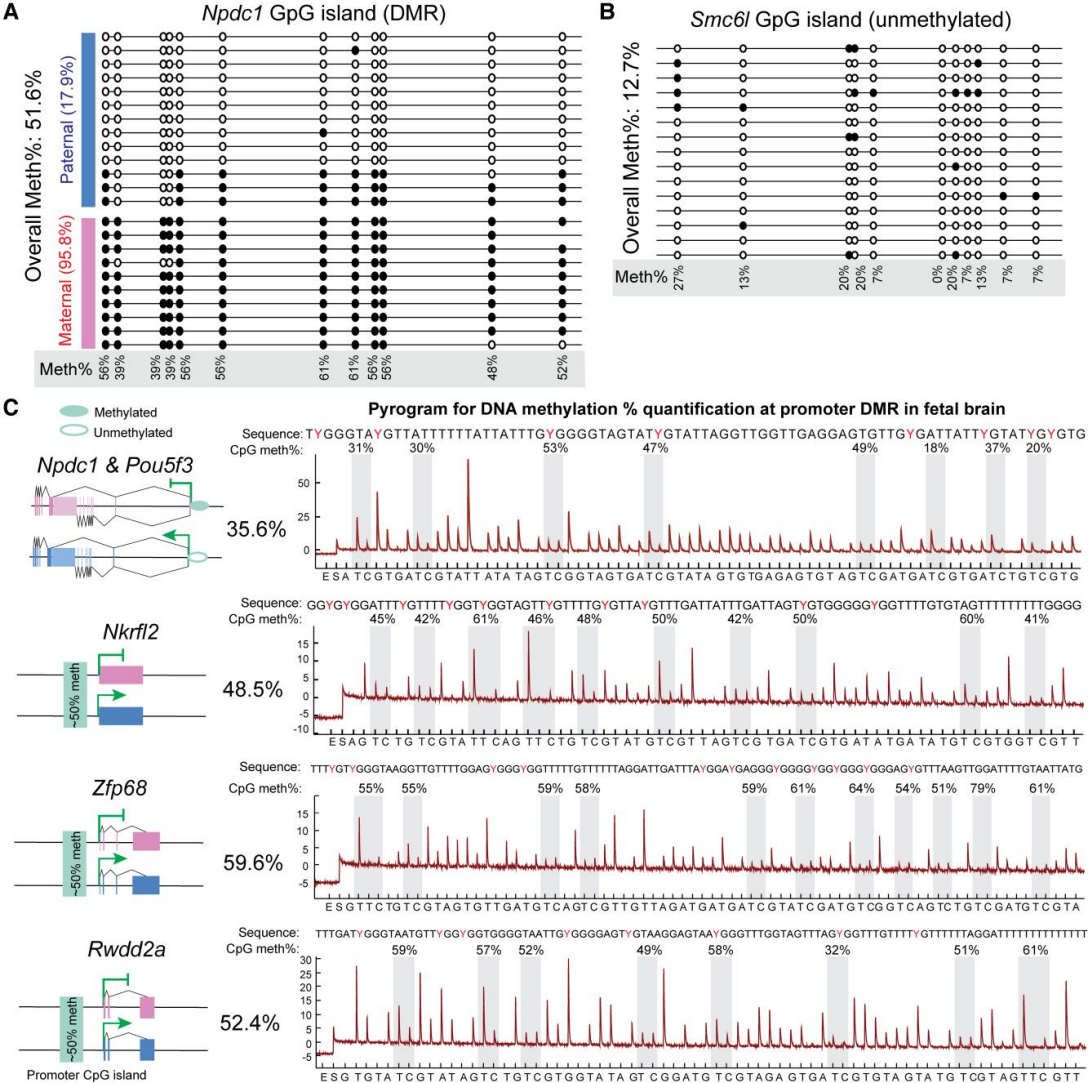
Promoter CpG islands DNA methylation profiling for *Smc6l*, *Npdc1-Pou5f3, Nkrfl2, Zfp68*, and *Rwdd2a.* (*A*) *Npdc1* promoter DNA methylation in fetal brain determined by cloning and Sanger sequencing after bisulfite treatment. The parental transmission direction was inferred from an information SNP between LL1 and LL2 strains in the amplified region. The solid and open circles represent methylated and unmethylated CpG sites, respectively. (*B*) Promoter DNA methylation profile for X-linked *Smc6l* in the fetal brain. (*C*) *Left*: gene regions of five confirmed imprinted genes with the maternal alleles in red and paternal alleles in blue. Right: programs and DNA methylation percentages at promoter CpGs quantified by PyroMark assays.

Differential methylation is an important epigenetic mechanism regulating genomic imprinting often seen in the promoter regions of eutherian imprinted loci. In *M. domestica*, differential methylation has only been detected in an intronic region of *Igf2r* (Das et al. 2012), but not in the promoter region. To determine the role of differential methylation in the marsupial imprinted genes reported here, we performed bisulfite treatment followed by cloning and Sanger sequencing, as well as PyroMark assays to quantify the DNA methylation percentages at the promoter CGIs (see Materials and Methods). Strikingly, we discovered differential DNA methylation in five protein-coding imprinted genes in both the fetal brain (fig. 4) and placenta (supplementary fig. S10, Supplementary Material online), indicating that these genes all harbor DMRs in their promoter regions. An informative SNP found between the LL1 and LL2 stocks in the *Npdc1* promoter was found to be associated with parent-of-origin-specific allele methylation (fig. 4*A*). The maternal promoter was completely methylated with an average DNA methylation of 96% over 12 CpGs (fig. 4*A*), whereas the paternal methylation was very low (18%), which is consistent with active transcription from the paternal allele (fig. 2*C*). Overall, the average methylation percentage at *Npdc1* promoter CGI was close to 50% (fig. 4*A*), which is the hallmark of a DMR. In contrast, the X-linked *Smc6l* had a nonmethylated promoter CGI (fig. 4*B*), in agreement with previous studies on the CGI methylation profiles for X-linked genes in *M. domestica* (Wang et al. 2014; Waters et al. 2018). CpG methylation percentages quantified by PyroMark assays and bisulfite sequencing identified the presence of allele-specific promoter DMR for *Npdc1* (fig. 4*A*and*C*)*. Pou5fl, Zfp68*, *Nkrfl2*, and *Rwdd2a* were shown by PyroMark to have approximately 50% methylation (35.6–59.6%) in the fetal brain (fig. 4*C*) and placenta (supplementary fig. S10, Supplementary Material online), suggesting the possibility of DMRs in these genes as well.

### Gene Identity of the Paternally Expressed POU Domain Transcription Factor

**Fig. 5.**
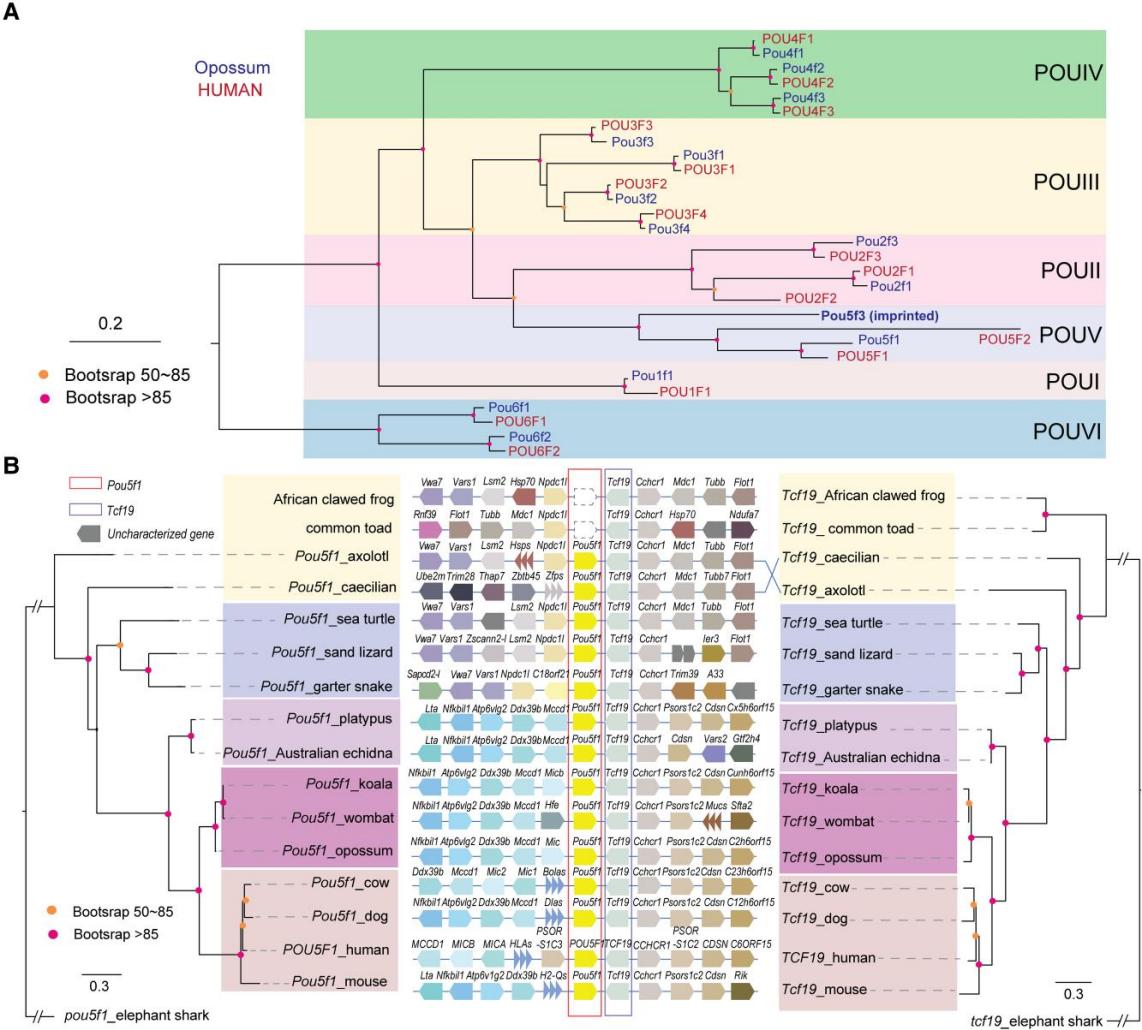
Genome neighborhood and phylogenetic analysis of *Pou5f1-Tcf19* in selected vertebrate species. (*A*) Phylogenetic tree of members in six POU domain families in *Monodelphis domestica* and *Homo sapiens*. (*B*) Maximum likelihood trees of protein sequences from *Pou5f1* (*left*) and its adjacent gene *Tcf19* (*right*), using their orthologs in elephant shark as the outgroup. Nodes with bootstrap values larger than 85% are labeled in red, and those between 50 and 85% are labeled in orange. The gene synteny in the genome neighborhood is shown in the *middle* panel, and dotted line boxes represent the loss of *Pou5f1* in African clawed frog and common toad.

One of the imprinted genes we discovered in *M. domestica, Pou5f3,* is a homeobox/POU domain transcription factor, which was not well annotated in the opossum reference genome (fig. 1). To determine gene identity and explore the evolutionary relationships of POU domain family members in *M. domestica*, we searched all paralogs and orthologs of POU genes in opossum and human genomes using BLASTp (see Materials and Methods). The majority of human POU genes, including *POU1F1*, *POU2F1*, *POU2F3*, *POU3F1*, *POU3F2*, *POU3F3, POU3F4*, *POU4F1*, *POU4F2*, *POU4F3, POU5F1, POU5F1,* and *POU6F1*, have an ortholog in the opossum (fig. 5*A*). *POU2F2* was not found in the opossum genome, but it is present in wallaby and koala genomes, suggesting that it may be missing from the monDom5 assembly. *POU5F2* was absent in all marsupial genomes we examined, and it may be human/eutherian-specific (fig. 5*A*). Our newly discovered imprinted gene, *Pou5f3*, is missing in the human genome. The closest human paralog, *Pou5f1,* also known as *Oct4* (Octamer-binding transcription factor 4), is one of the four transcription factors for establishing and maintaining pluripotency (Takahashi and Yamanaka 2006; Yu et al. 2007). In zebrafish, *Pou5f1* is missing, and *Pou5f3* (gene ID: ZDB-GENE-980526-485) is the only copy of *Oct4* (Palfy et al. 2020). We checked the expression and allelic imbalance of all genes in the six POU domain families in our *M. domestica* RNA-seq dataset and found *Pou3f1, Pou3f2, Pou4f1,* and *Pou6f1* were moderately expressed in fetal brain and exhibited biallelic expression. *Pou1f1* had much lower expression in brain, and the RNA-seq reads were observed from both alleles. *Pou5f1* was biallelically expressed in placenta. As expected, X-linked *Pou3f4* showed monoallelic expression from the maternal allele due to imprinted X inactivation in *M. domestica* (Douglas 2013). Expression was not detected for the remaining POU genes in our RNA-seq data. Therefore, *Pou5f3* is the only paternally expressed autosomal imprinted gene among the POU domain transcription factors in *M. domestica*.

### Evolution of *Pou5f3-Npdc1* Imprinting Cluster in Vertebrate Lineages

**Fig. 6.**
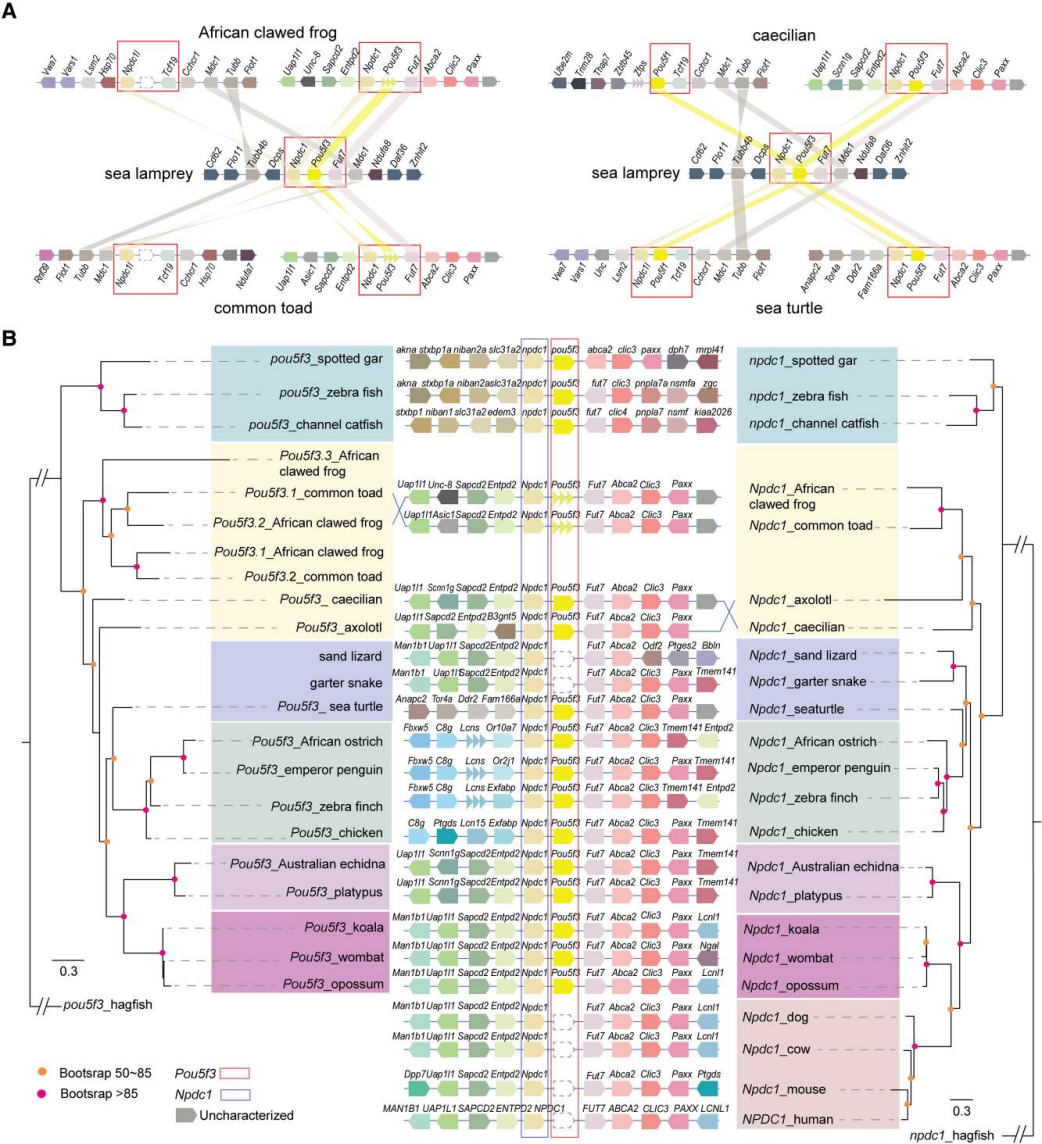
Genome neighborhood and phylogenetic analysis of *Pou5f3-Npdc1* in selected vertebrate species. (*A*) Comparative synteny analysis of the *Npdc1*-*Pou5f3*-*Fut7* loci between sea lamprey and other vertebrates (African clawed frog, common toad, caecilian, and sea turtle). (*B*) Maximum likelihood trees of protein sequences from *Pou5f3* (*left*) and *Npdc1* (*right*), using orthologs in hagfish as the outgroup. Nodes with bootstrap values larger than 85% are labeled in red, and those between 50 and 85% are labeled in orange. Synteny in the genome neighborhood is shown in the *middle* panel, and dotted line boxes represent gene loss.

Frankenberg and Renfree found that *Pou5f1* and *Pou5f3* (designated POU2 in the Frankenberg and Renfree study) are derived from an ancestral duplication event (Frankenberg and Renfree 2013). Our phylogenetic and comparative synteny analyses are in general agreement with Frankenberg and Renfree, but also reveal greater detail concerning the complexity of evolution in the *Pou5f1*/*Pou5f3* region. Vase tunicate (*Ciona intestinalis*) only has POU class 2 (XP_009858287.1) and class 4 transcription factors (NP_001027972.1) in its genome (assembly GCF_000224145.3). The lack of *Pou5f1*/*Pou5f3* in tunicates suggested that POUV is vertebrate-specific. The jawless fish sea lamprey (*Petromyzon marinus*) has a single copy of *Pou5f3* flanked by *Npdc1* and *Fut7*, reflecting the ancestral status of the POUV locus (fig. 6*A*). In the common ancestor of Gnathostomata, a duplication event at the *Npdc1*-*Pou5f3*-*Fut7* locus occurred, resulting in the new *Npdc1l*-*Pou5f1* locus downstream of *Tcf19* (fig. 6*A*) (Frankenberg and Renfree 2013). Subsequently, one of the two POUV genes was lost in most vertebrates, with a few exceptions (figs. 5–6). Bony fish, birds, and some amphibians (African clawed frog and common toad) lost *Pou5f1* (fig. 5), and they utilize *Pou5f3* as the *Oct4* gene in these lineages (fig. 6). In contrast, *Pou5f3* is missing, and *Pou5f1* is retained in the genomes of some reptiles (sand lizard and garter snake) and eutherian mammals (fig. 6*B*). Certain amphibians and reptiles, including axolotl, two-lined caecilian, and sea turtles, retained both *Pou5f1* and *Pou5f3* in their genome (figs. 5–6). Notably, all monotremes and marsupial species we examined have both *Pou5f1* and *Pou5f3* copies, including the platypus, Australian echidna, opossum, wombat, and koala (figs. 5–6). The evolution of the *Pou5f1*/*Pou5f3* loci was complicated by rapid turnover of the genes in the neighborhood (fig. 6). Tandem duplication of three copies of *Pou5f3* was detected in the African clawed frog and common toad (fig. 6). The direction of *Fut7* was reversed in the common ancestor of therian mammals. The *Npdc1l* copy upstream of *Pou5f1* was lost in the mammalian common ancestor (fig. 5). Although these genes have maintained a high degree of synteny generally, the composition, copy number, and direction of certain neighboring genes are altered in many species that we investigated (figs. 5–6).

### The PEGs, *Nkrfl1* and *Nkrf2*, Originated by a Marsupial-specific duplication

**Fig. 7.**
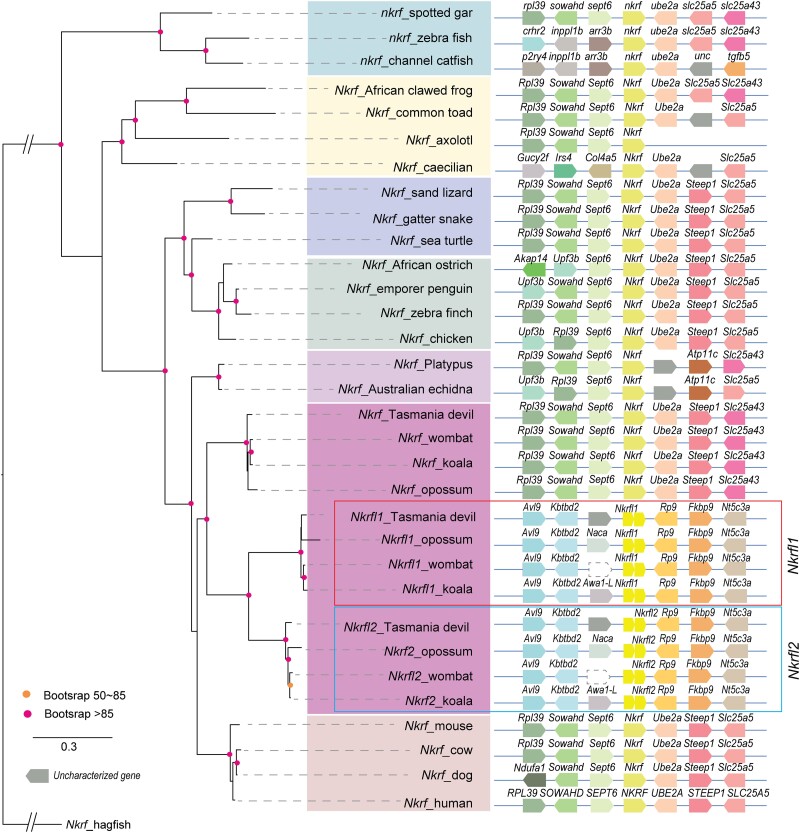
Genome neighborhood and phylogenetic analysis of *Nkrf* and its paralog *Nkrfl* in selected vertebrate species. Maximum likelihood trees were constructed using protein sequences from *Nkrf* and *Nkrfl* (*left*) using their ortholog in hagfish as the outgroup. Nodes with bootstrap values larger than 85% are labeled in red and those between 50 and 85% are labeled in orange. Synteny in the genome neighborhood is shown in the *right* panel. Dotted line boxes represent gene loss events.

We discovered that *Nkrfl1* and *Nkrfl2* are PEGs in *M. domestica.* They are paralogs of *Nkrf*, nuclear factor kappa B repressing factor, which is a negative regulator of NF kappa B responsive genes (Nourbakhsh and Hauser 1999) located in nucleoli (Niedick et al. 2004). *Nkrf* is X-linked in marsupials and eutherians, and we identified its orthologs in 20 additional vertebrate species (fig. 7; see Materials and Methods). A single copy of *Nkrf* is found in fish, amphibians, reptiles, birds, monotremes, and eutherian mammals (fig. 7), but three homologs were found in the marsupial species we examined. We propose that in the common ancestor of marsupials, an X-autosome retroposition event occurred, followed by a tandem duplication, resulting in *Nkrfl1* and *Nkrfl2* on the ancestral chromosome that became chromosome 6 in modern *M. domestica* (fig. 7).

## Discussion

### Marsupials Imprint a Smaller set of Genes Compared With Eutherian Mammals

Genomic imprinting was first discovered in mice (Tucci et al. 2019), and extensive subsequent research, mostly in humans and mice, has shown that imprinted loci comprise 1–2% of eutherian genes (Luedi et al. 2005; Luedi et al. 2007). A few orthologs of these known eutherian imprinted genes have been investigated in marsupials, and some of them were confirmed to be imprinted in these species (Killian et al. 2000; O'Neill et al. 2000; Suzuki et al. 2005; Weidman et al. 2006; Ager et al. 2007; Suzuki et al. 2007; Smits et al. 2008; Das et al. 2012; Edwards et al. 2019). However, due to the attendant ascertainment bias toward eutherian imprinted genes using this approach, such results are not sufficient to reach strong conclusions regarding the conservation of genomic imprinting profiles between marsupials and eutherian mammals generally. To counter this shortcoming, we performed an unbiased survey of imprinted genes in opossums and identified 13 autosomal imprinted genes. Since our informative SNPs only covered ∼20% of all known expressed genes in opossum, we estimate that ∼60 genes in the *M. domestica* autosomal genome are imprinted, which is a much smaller number by comparison with eutherian mammals. In addition, 75% of the imprinted genes we detected are novel and not known to be imprinted in any other species, suggesting evolutionary fluidity of genomic imprinting between marsupials and eutherians. The list of genes exclusively imprinted in the opossum helps to fill some of the gaps in our knowledge of the genomic imprinting profile in marsupials.

### Lack of Tissue Specificity Between the Fetal Brain and Placenta in the Marsupial Imprinting Profile

The brain and placenta are hotspots for imprinted genes, and a survey of 82 imprinted genes in mice revealed substantial tissue specificity of genomic imprinting, with 28% of imprinted genes strictly imprinted in only one tissue (Prickett and Oakey 2012). However, tissue specificity was not observed in our opossum data. Among the 10 novel imprinted genes we identified, eight were imprinted in both the fetal brain and placenta, while the imprinting status for the remaining two genes could not be determined in the placenta due to insufficient expression levels. The remarkably similar imprinting profiles in brain and placenta mirrors the escaping profile for imprinted X chromosome inactivation (XCI) in opossum brain and placenta, which also lacks tissue specificity (Wang et al. 2014). In contrast, the XCI escaping profiles in the placenta and adult tissues are completely different in eutherian mammals, with almost no overlap in the escaping gene sets (Phung et al. 2022). We speculate that the major differences between marsupial and eutherian imprinting and XCI pattern could be a result of differences in tissue origins in early embryonic development. In the late eutherian blastocyst stage, internal cells known as the inner cell mass (ICM) are destined to develop into the embryo proper, whereas the outer trophoblast layer cells give rise to the placenta (Gilbert 2010). The mouse brain and placenta have different imprinting and XCI profiles due in part to the differential timing of XCI in the embryonic cell type in which the X chromosome is inactivated. In contrast, the marsupial embryo and yolk sac placenta are both derived from the trophectoderm (Mate et al. 1994; Zeller and Freyer 2001; Selwood and Johnson 2006), likely subsequent to the initiation of XCI, and possibly after somatic cell imprinting has occurred as well. If so, the absence of spatially distinct ICM and trophectoderm compartments may explain the similarity of imprinting profiles in the embryo and its EEM-derived structures in opossum.

### Conserved Epigenetic Regulatory Mechanisms of Genomic Imprinting in Marsupials—Maternal Allele-specific Methylation is Associated With Paternal Allele-specific Expression

The molecular mechanisms of genomic imprinting have been extensively studied in eutherian mammals. Differential DNA methylation, histone modification, and regulatory noncoding RNA are the most important mechanisms driving monoallelic expression at imprinted loci (Bartolomei and Ferguson-Smith 2011). In this study, we focused on searching for DMRs at the promoters of the novel imprinted genes we discovered. The effects of CpG methylation on gene expression depend on the genomic context in which the gene occurs. Promoter DNA methylation is often associated with gene silencing, whereas DNA methylation at CTCF-containing enhancer blocker regions prevents CTCF binding and activates gene expression in *cis*. In the opossum, an imprinted DMR has heretofore been observed only in an intron of *Igf2r* (Das et al. 2012). Since more than one-third of eutherian imprinted genes have an associated DMR, we examined promoter CGI methylation in each of our newly discovered opossum imprinted genes with promoter annotation, that is the protein-coding genes (the noncoding RNAs lack appropriate annotation to determine the physical location of promoter CGIs). Five of the opossum imprinted genes (*Npdc1*, *Pou5f3*, *Nkrfl*, *Zfp68*, and *Rwdd2a*) have ∼50% DNA methylation (35.6–59.6%), suggesting the possibility of DMRs at promoter CGIs and warrant further confirmation using DNA methylome analysis with genome-wide coverage. Due to the lack of informative SNPs, only one gene (*Npdc1*) was confirmed to have a parent-of-origin-specific DMR. These five novel imprinted genes, together with the X-linked imprinted gene *Rsx* (Grant et al. 2012), whose promoter DMR was identified by our previous research (Wang et al. 2014), are all PEGs. Maternally expressed genes in the opossum include *Meis1* on chromosome 1 and ∼300 X-linked genes that are subject to imprinted XCI, whose promoter CGIs are nonmethylated (Douglas et al. 2014; Wang et al. 2014; Waters, et al. 2018). This is consistent with previous eutherian findings, that the majority of eutherian ICRs are maternally methylated, with only three intergenic paternally methylated ICRs. This pattern of biased promoter methylation on the maternal allele suggests distinct epigenetic regulatory mechanisms for paternal versus maternal monoallelic expression in marsupials, which could be due to differential epigenetic reprogramming in male versus female germlines.

### Toward a Comprehensive Genomic Imprinting Profile for Marsupials

We quantified allelic expression in reciprocal crosses of two opossum strains to detect genomic imprinting in an unbiased way. Although we discovered nine novel imprinted genes and revealed promoter differential DNA methylation as an important epigenetic mechanism in marsupial imprinting, our study is likely to have failed to detect many imprinted genes. First, the LL1 and LL2 strains we used are random-bred stocks, and LL2 has ancestral admixture from LL1, resulting in shared segregating polymorphisms between them. As a consequence, we were only able to cover ∼20% of expressed genes with informative SNPs, which reduced our ability to achieve a truly genome-wide survey. Second, the *Igf2*-*H19* cluster, a major imprinting center in human and mouse, was not assembled in the opossum reference genome. We addressed this issue by de novo transcript assembly, but the non-transcribed regulatory regions are still missing. Third, we performed targeted DNA methylation assays for selected imprinted genes. DNA methylome profiles in F1 crosses would be more informative in detecting DMRs on a genome-wide basis. For a systematic comparison of marsupial and eutherian genomic imprinting profiles, we will need an improved *M. domestica* reference genome, unrelated inbred strains to track the parental transmission direction and achieve genome-wide coverage, and epigenomic assays to reveal the mechanisms of marsupial imprinting.

### The Pluripotency Factor *Oct4* has two Copies in Marsupials (*Pou5f1* and *Pou5f3*), and one is Imprinted in the Opossum

Oct4 is one of the core transcription factors which, together with Sox2 and Nanog, maintain the pluripotent state of ESCs (embryonic stem cells) (Young 2011). The generation of induced pluripotent stem cells (iPSCs) requires the expression of *Oct4* and *Sox2* (Stadtfeld and Hochedlinger 2010). In eutherian mammals, the core pluripotency factors are largely conserved. Gene expression analysis on iPSCs from the Tasmanian Devil also showed this to be the case in marsupial mammals (Weeratunga et al. 2018). There is substantial complexity in the evolutionary history of *Oct4*. Two paralogous copies originated in jawed vertebrates through a duplication event, resulting in *Pou5f1* and *Pou5f3*. The subsequent loss of one paralog has occurred in most vertebrate lineages, but the marsupial clade has retained both *Pou5f1* and *Pou5f3*, suggesting that these paralogs may have important individual or complementary functions. A recent study utilizing *M. domestica* inbred strains established and validated iPSCs in a marsupial model for the first time (Kumar et al. 2022) and revealed that both *Pou5f1* and *Pou5f3*, as well as their splice variants, are expressed in different cell lineages and reprogrammed *M. domestica* iPSCs during embryonic development and organ development. Transcriptome analysis revealed that the core pluripotency gene network and the functional profile of the *M. domestica* iPSCs are strongly similar to eutherians, indicating highly conserved regulatory mechanisms (Kumar et al. 2022). The *Pou5f3* splice variant was found to play a synergistic role with *Pou5f1* in the regulation of the opossum pluripotency gene network. Since *Oct4* expression levels control the pluripotency of stem cells in a quantitative manner (Niwa et al. 2000), tight regulation of *Pou5f1* and *Pou5f3* expression is indispensable for proper differentiation, and imprinting of *Pou5f3* in *M. domestica* might play a crucial role in finer regulation of its expression in early development. We speculate that after the evolutionary origin of *Pou5f3* imprinting, paternal monoallelic expression was maintained by the establishment of stable differential methylation on the maternal allele.

### Evolutionary Origin of Marsupial Imprinted Genes—Imprinted Noncoding RNAs

The marsupial-specific imprinted genes discovered by this study provide hints for the evolutionary origin of species-specific genomic imprinting. In almost every eutherian imprinting cluster, at least one noncoding RNA with clear regulatory functions is present. We believe that our study is the first to identify imprinted noncoding RNA in marsupials, including *Ipncr2*, which is adjacent to a protein-coding imprinted genes *Nkrfl1* and *Nkrfl2*. The three imprinted noncoding RNAs (*Ipncr1*, *Ipncr2*, and *Ipncr5*) lack orthology in eutherian mammals and, conversely, many eutherian imprinted noncoding RNAs cannot be identified in marsupials, suggesting rapid birth and death of imprinted noncoding RNAs.

### Evolutionary Origin of Marsupial Imprinted Genes—Lineage-specific Gene Duplication and Retrotransposition

The paternally expressed imprinted gene *Pou5f3* is a paralog of *Pou5f1*, the pair having resulted from an ancient duplication event. Most vertebrates only retain one copy of *Oct4* family genes, but marsupials retain both paralogs, with *Pou5f3* exhibiting imprinted expression in both fetal brain and placenta of opossum, and *Pou5f1* being biallelically expressed in the placenta. Another paternally expressed imprinted cluster in opossum contains tandemly duplicated genes *Nkrfl1* and *Nkrfl2*, which were derived from retrotransposition from the X-linked gene *Nkrf* in the common ancestor of marsupials. Interestingly, retrotransposed copies of X-linked genes were also identified to be imprinted with paternal expression in eutherian mammals, including *Mcts2*, *Nap1l5*, *U2af1-Rs1*, *Inpp5f_v2,* and *Peg12* in mice (Wood et al. 2007; Cowley and Oakey 2010). Our findings in opossum have broadened the lineage spectrum, suggesting that there is a shared evolutionary mechanism underlying the imprinting of transposed X-linked genes in both eutherian and metatherian mammals. The foregoing observations indicate that marsupial-specific duplicated genes may be regulated in a way that can eventually favor stable parent-of-origin-specific expression. If so, this might be explained by the dosage-sharing hypothesis (Lan and Pritchard 2016), which proposes that tandemly duplicated genes have a reduction in gene expression to match the level of single-copy genes. Genomic imprinting is a mechanism that can reduce gene expression level by inhibiting one of the two parental alleles, in a developmental stage-specific manner, which could partially compensate for a potentially toxic dose of duplicated genes.

### Genetic Conflict and Imprinting in Marsupials?

Inasmuch that our study only interrogated ∼20% of the expressed genes in the opossum transcriptome, our results are not sufficient to formally test the genetic conflict hypothesis (Moore and Haig 1991). In addition, empirical data on most gene functions are lacking for the opossum, and functional annotation of the genome assembly is mostly inferred by homology. The newly discovered, marsupial-specific imprinted genes described herein do not appear to have obvious growth effects; however, their functions are pivotal during early embryonic development. *Pou5f3*/*Oct4* is the POU domain-containing transcription factor, which participates in chromatin structure remodeling at zygotic genome activation (Veil et al. 2019). The NF-κB repressing factor (*Nkrfl*) plays a critical role in maintaining nucleolar homeostasis by preventing aberrant precursors during rRNA processing (Coccia et al. 2017). *Npdc1* regulates terminal neural differentiation, and its mRNA is a target of m(6)A methylation (Zhou et al. 2021). The Krab zinc finger protein *Zfp68* was shown to be a transcription repressor, which helps establish the silencing histone mark H3K9me3 in partnership with *Trim28* (Mun 2021). The functions of these novel imprinted genes suggest that the developmental plasticity hypothesis might be a plausible model for the evolution of genomic imprinting in the opossum, and in marsupials generally (Radford et al. 2011). Additional research is needed to obtain the comprehensive imprinting profile for all genes to further test various hypotheses regarding the evolutionary origins of genomic imprinting.

## Supplementary Material

msad022_Supplementary_Data

## Data Availability

The raw RNA-seq data and gene read counts are available at NCBI GEO (Gene Expression Omnibus) databases under the accession number GSE45211.
