# Supplementary material for: Origin and Evolution of Marsupial-specific Imprinting Clusters Through Lineage-specific Gene Duplications and Acquisition of Promoter Differential Methylation
Source: Mol Biol Evol. 2023 Jan 31;40(2):msad022. doi: 10.1093/molbev/msad022 (PMC9937046; doi:10.1093/molbev/msad022)

Figure S1. Parent-of-origin allelic expression ratios of an imprinted gene *Igf2r* in *Monodelphis domestica* fetal brain and placenta.

(**A**) *Igf2r* SNP genotypes in parental and F1 animals determined by Sanger sequencing in LL1 (dam) X LL2 (sire) and reciprocal LL2 X LL1 crosses. NI, Not Informative.

(**B**) *Igf2r* differential allelic expression profile estimated from RNA-seq and validated by allele-specific pyrosequencing in the two reciprocal crosses. The maternal allelic percentages are shown in red and paternal percentages are shown in blue.

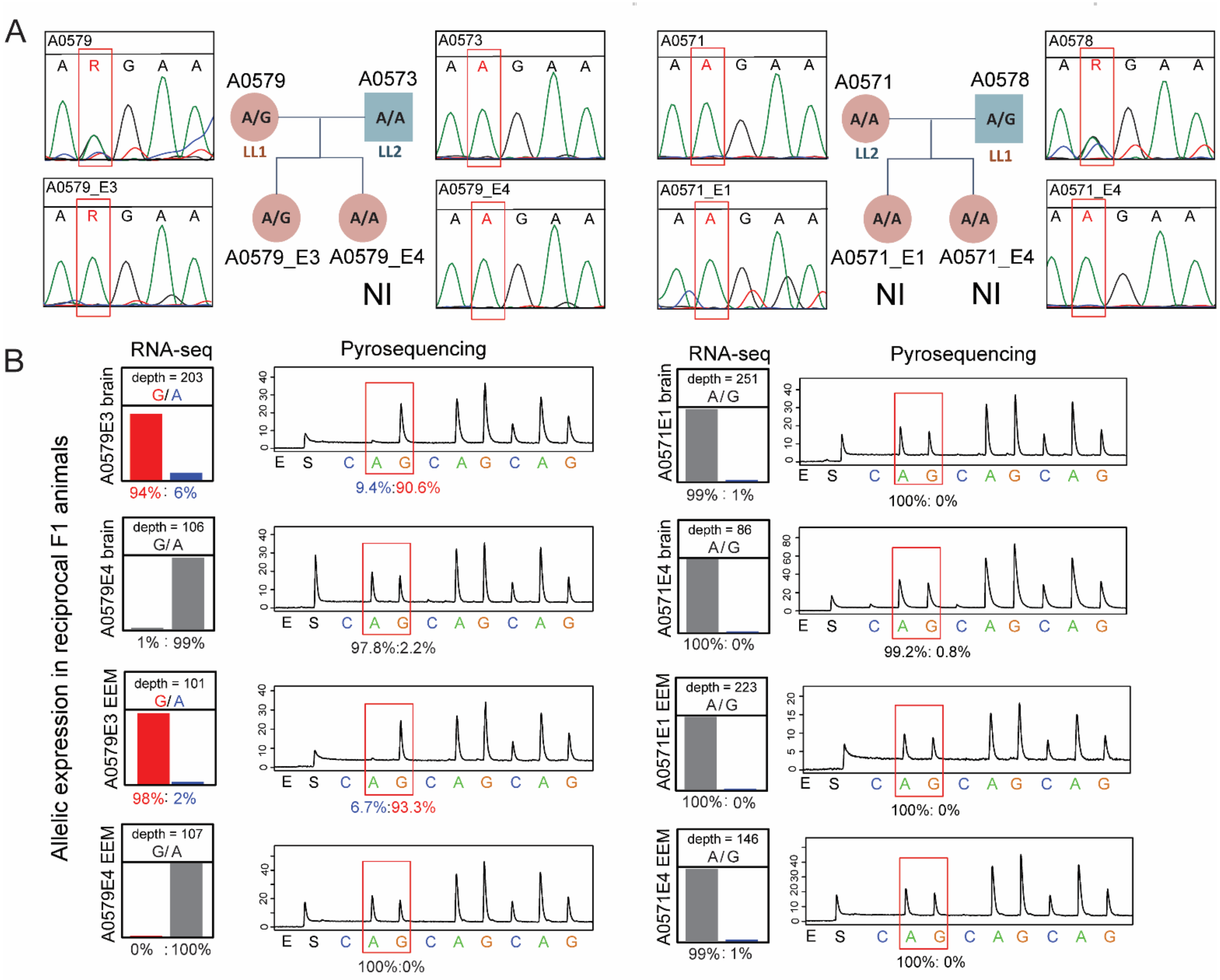

Figure S2. Parental and F1 sample SNP genotypes for noninformative genes *Matn2*, *Prkaa2, and Parp4* determined by Sanger sequencing.

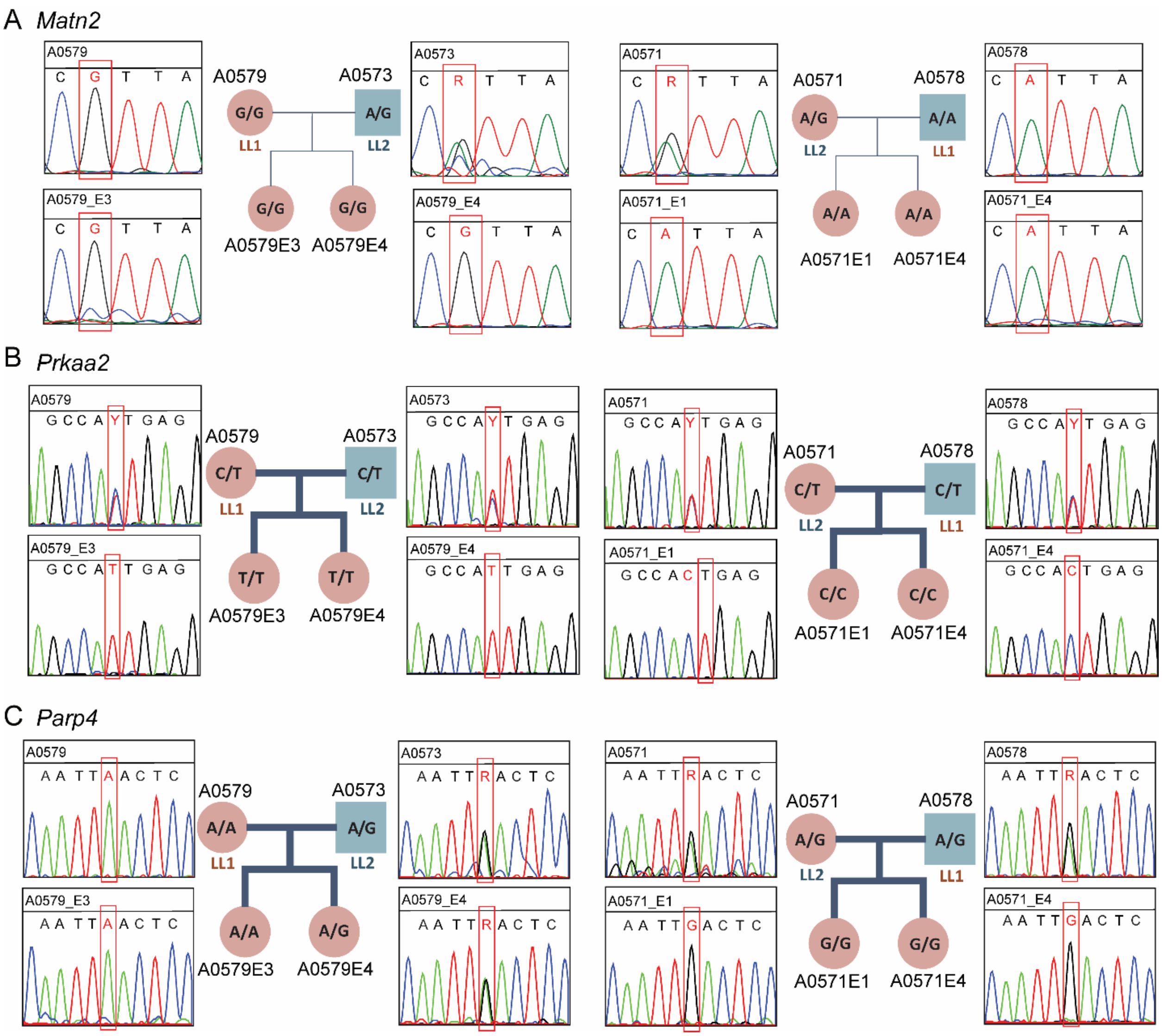

Figure S3. Parent-of-origin allelic expression ratios of an imprinted gene *Pou5f3* in *Monodelphis domestica* fetal brain and placenta.
(A) *Pou5f3* SNP genotypes in parental and F1 animals determined by Sanger sequencing, in LL1 (dam) X LL2 (sire) and reciprocal LL2 X LL1 crosses.
(B) *Pou5f3* differential allelic expression profile estimated from RNA-seq and validated by allele-specific pyrosequencing in the two reciprocal crosses. The maternal allelic percentages are shown in red and paternal percentages are shown in blue.

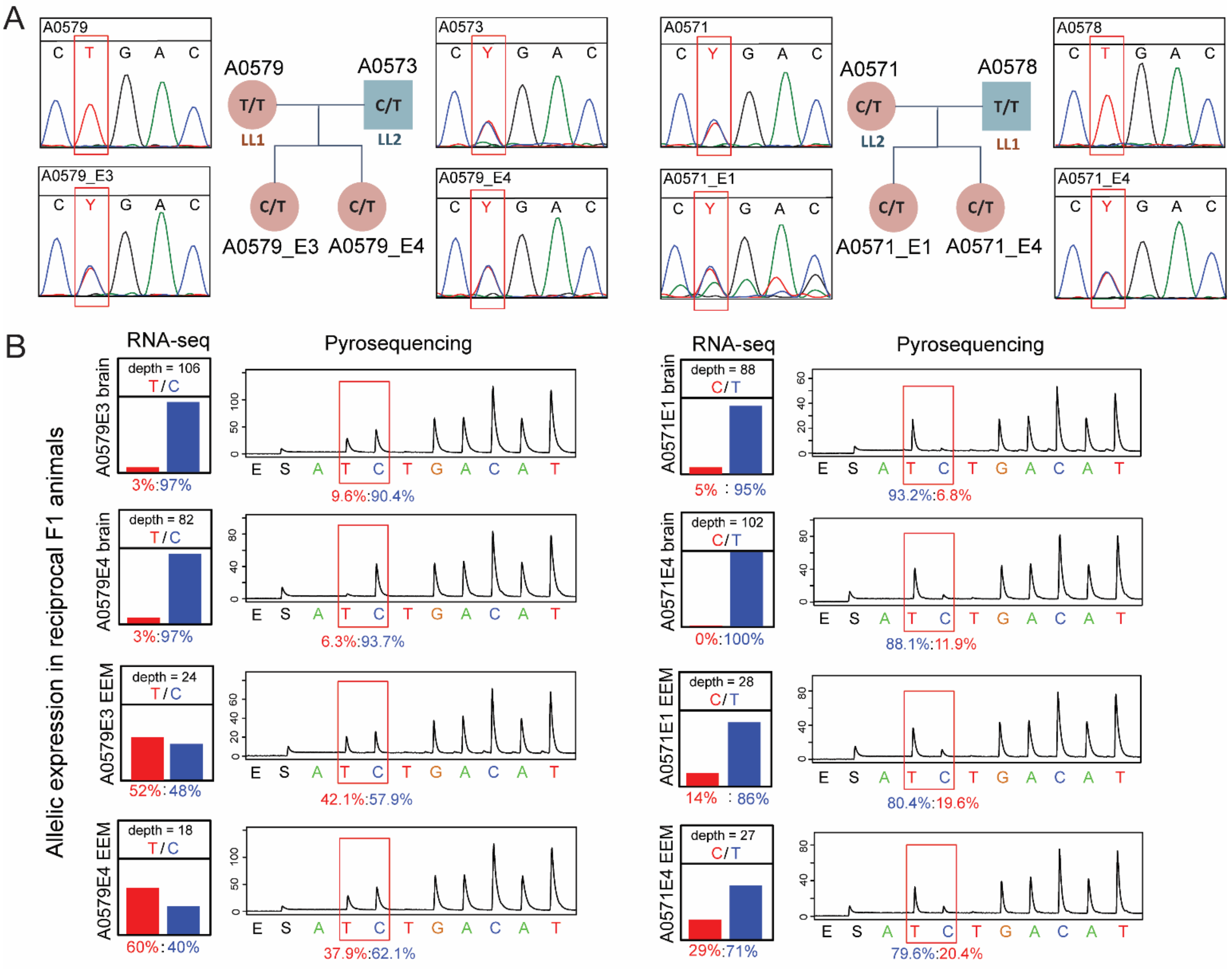

Figure S4. Parent-of-origin allelic expression ratios of a novel imprinted gene *Ipncr2* in *Monodelphis domestica* fetal brain and placenta.
(**A**) *Ipncr2* SNP genotypes in parental and F1 animals determined by Sanger sequencing in LL1 (dam) X LL2 (sire) and reciprocal LL2 X LL1 cross.
(**B**) *Ipncr2* differential allelic expression profile estimated from RNA-seq and validated by allele-specific pyrosequencing in the two reciprocal crosses. The maternal allelic percentages are shown in red and paternal percentages are shown in blue.

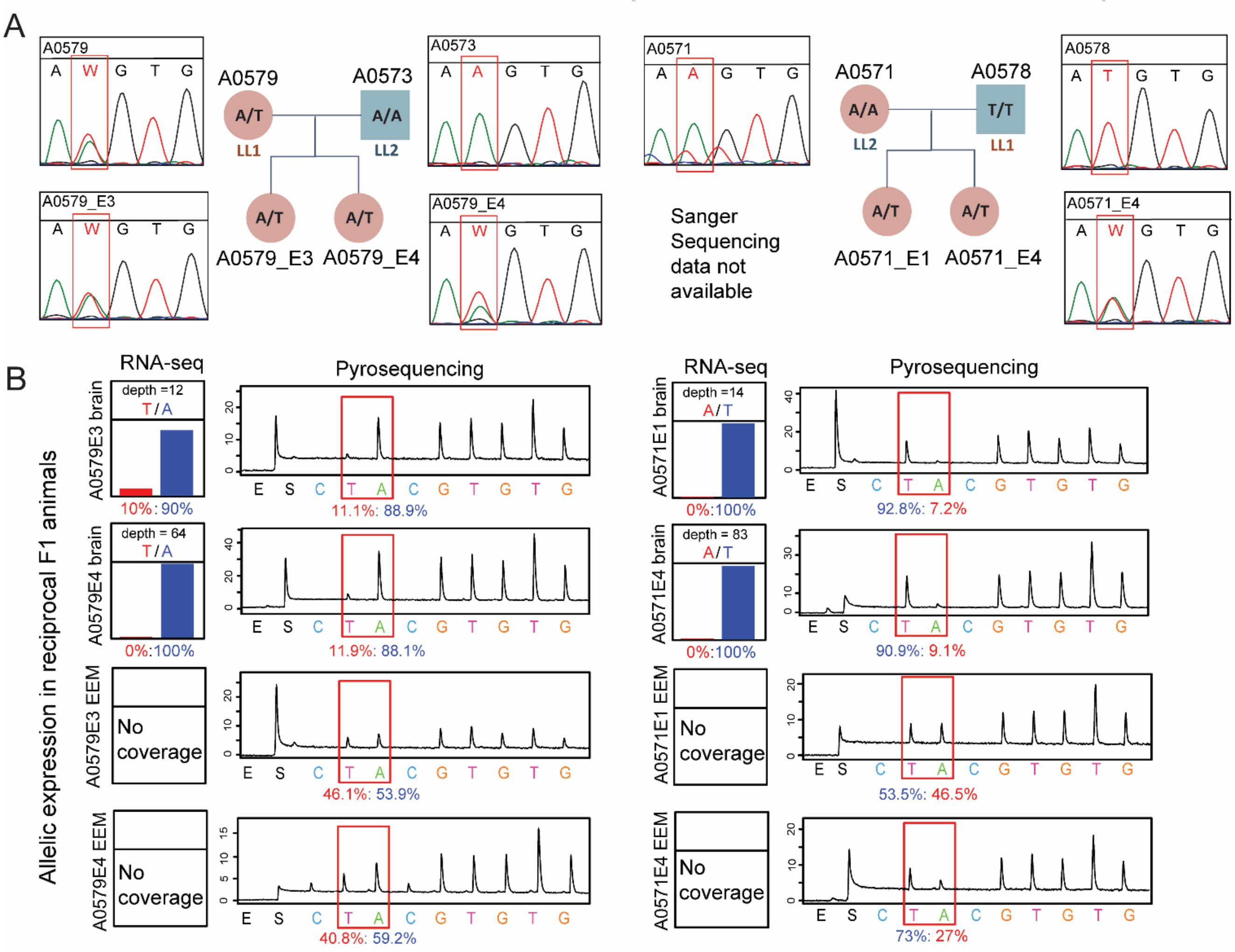

Figure S5. Parent-of-origin allelic expression ratios of a novel imprinted gene *Ipncr1* in *Monodelphis domestica* fetal brain.

(**A**) *Ipncr1* SNP genotypes in parental and F1 animals determined by Sanger sequencing in LL1 (dam) X LL2 (sire) and reciprocal LL2 X LL1 crosses.

(**B**) *Ipncr1* differential allelic expression profile estimated from RNA-seq and validated by allele-specific pyrosequencing in the two reciprocal crosses. The maternal allelic percentages are shown in red and paternal percentages are shown in blue.

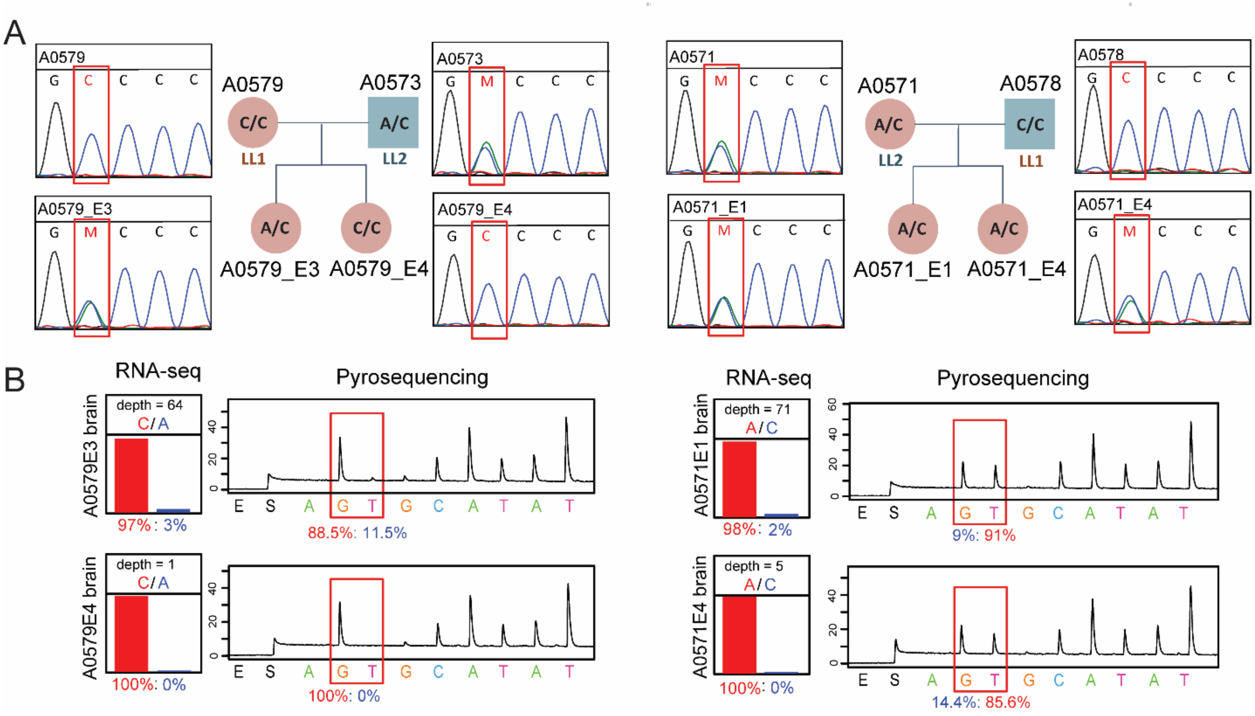

Figure S6. Parent-of-origin allelic expression ratios of an imprinted gene *Ipncr5* in *Monodelphis domestica* fetal brain and placenta.

(**A**) *Ipncr5* SNP genotypes in parental and F1 animals determined by Sanger sequencing in LL1 (dam) X LL2 (sire) and reciprocal LL2 X LL1 crosses. NI, Not Informative.

(**B**) *Ipncr5* differential allelic expression profile estimated from RNA-seq and validated by allele-specific pyrosequencing in the two reciprocal crosses. The maternal allelic percentages are shown in red and paternal percentages are shown in blue.

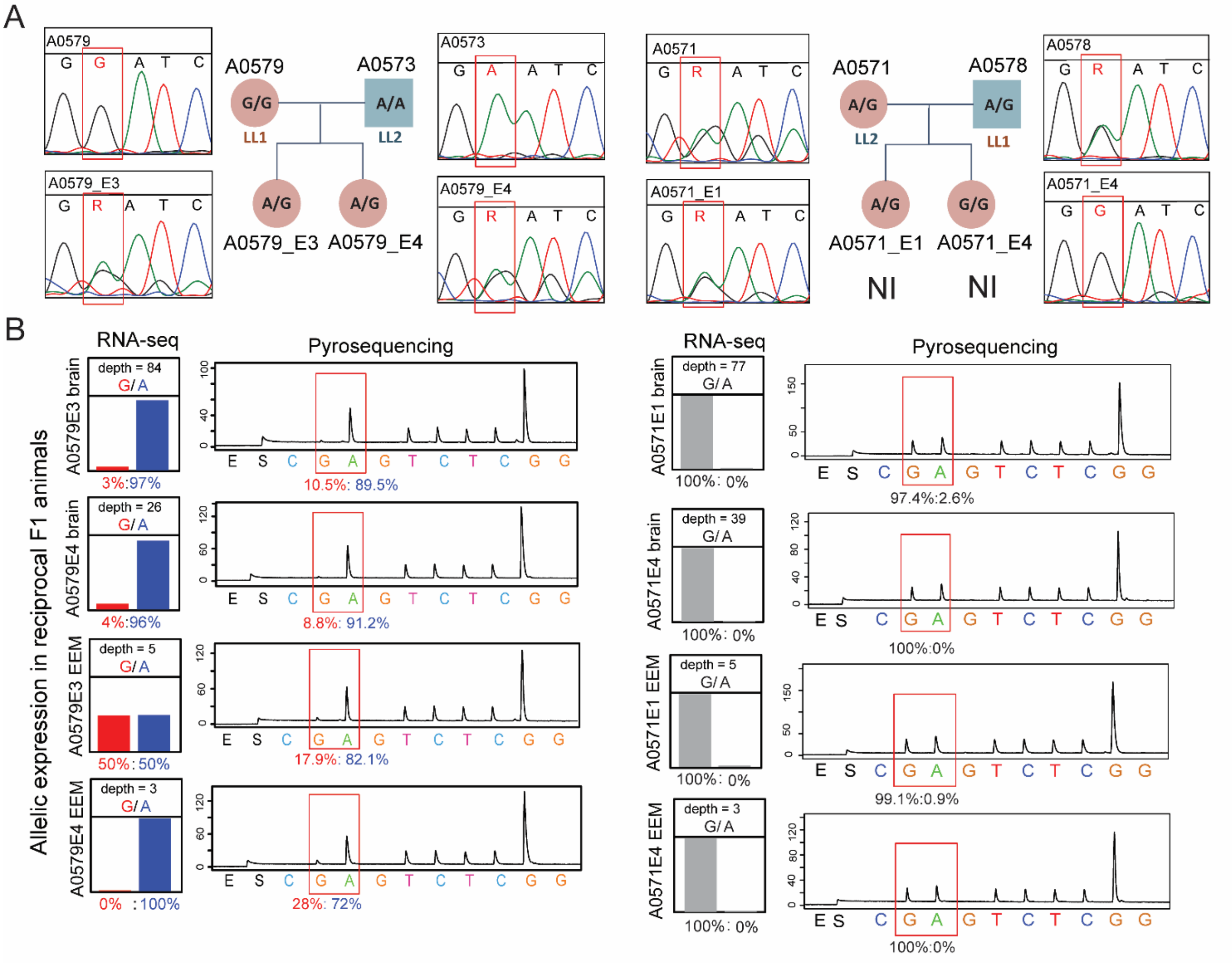

Figure S7. Parent-of-origin allelic expression ratios of an imprinted gene *Rwdd2a* in *Monodelphis domestica* fetal brain and placenta).

(A) *Rwdd2a* SNP genotypes in parental and F1 animals determined by Sanger sequencing in LL1 (dam) X LL2 (sire) and reciprocal LL2 X LL1 crosses. NI, Not Informative.

(B) *Rwdd2a* differential allelic expression profile estimated from RNA-seq and validated by allele-specific pyrosequencing in the two reciprocal crosses. The maternal allelic percentages are shown in red and paternal percentages are shown in blue.

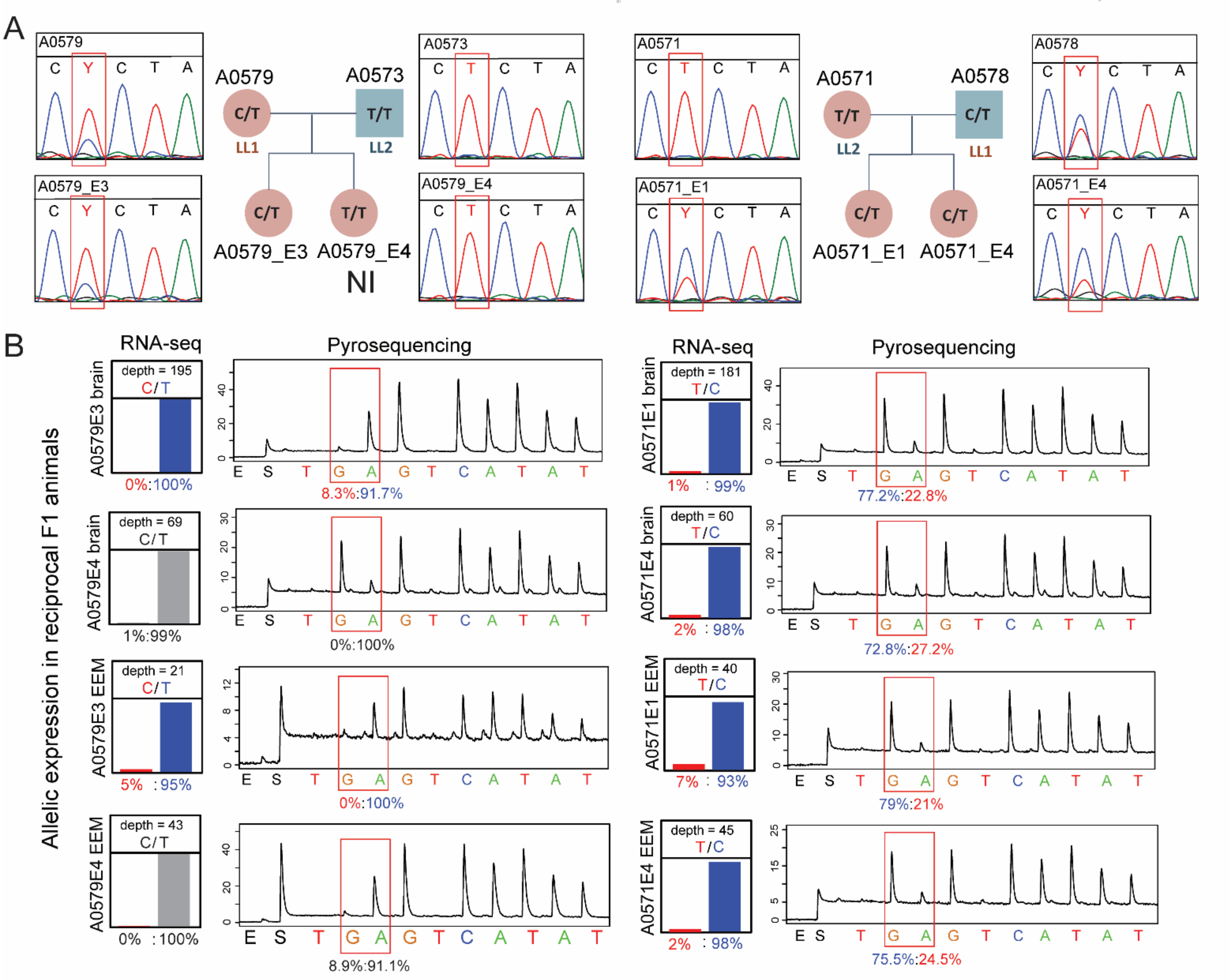

Figure S8. Parent-of-origin allelic expression ratios of an imprinted gene *Zfp68* in *Monodelphis domestica* fetal brain and placenta.
(**A**) *Zfp68* SNP genotypes in parental and F1 animals determined by Sanger sequencing in LL1 (dam) X LL2 (sire) and reciprocal LL2 X LL1 crosses. NI, Not Informative.
(**B**) *Zfp68* differential allelic expression profile estimated from RNA-seq and validated by allele-specific pyrosequencing in the two reciprocal crosses. The maternal allelic percentages are shown in red and paternal percentages are shown in blue.

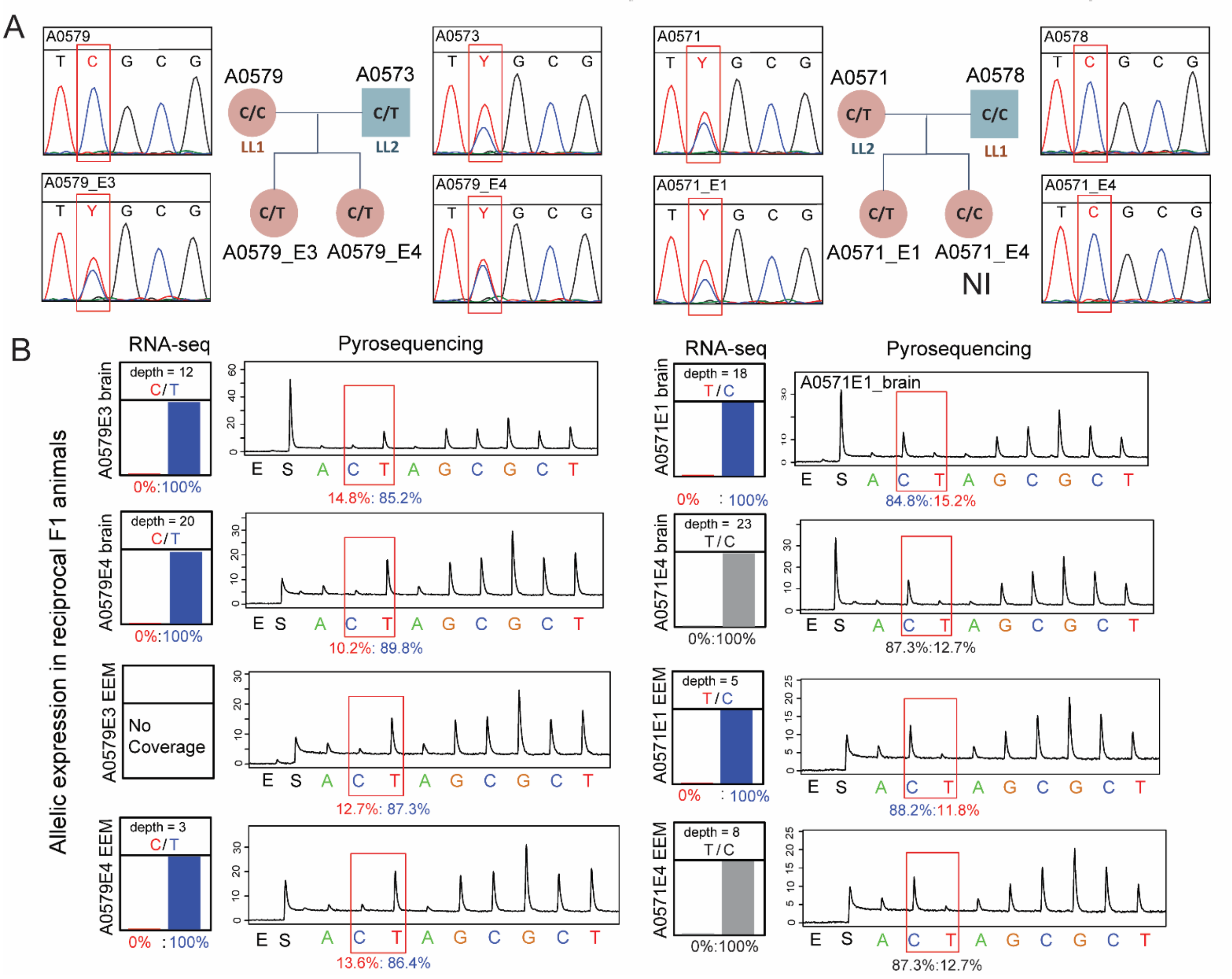

Figure S9. Parent-of-origin allelic expression ratios of an imprinted gene *Fam169a* in *Monodelphis domestica* fetal brain and placenta.
(**A**) *Fam169a* SNP genotypes in parental and F1 animals determined by Sanger sequencing in LL1 (dam) X LL2 (sire) and reciprocal LL2 X LL1 crosses. NI, Not Informative.
(**B**) *Fam169a* differential allelic expression profile estimated from RNA-seq in the two reciprocal crosses. The maternal allelic percentages are shown in red and paternal percentages are shown in blue.

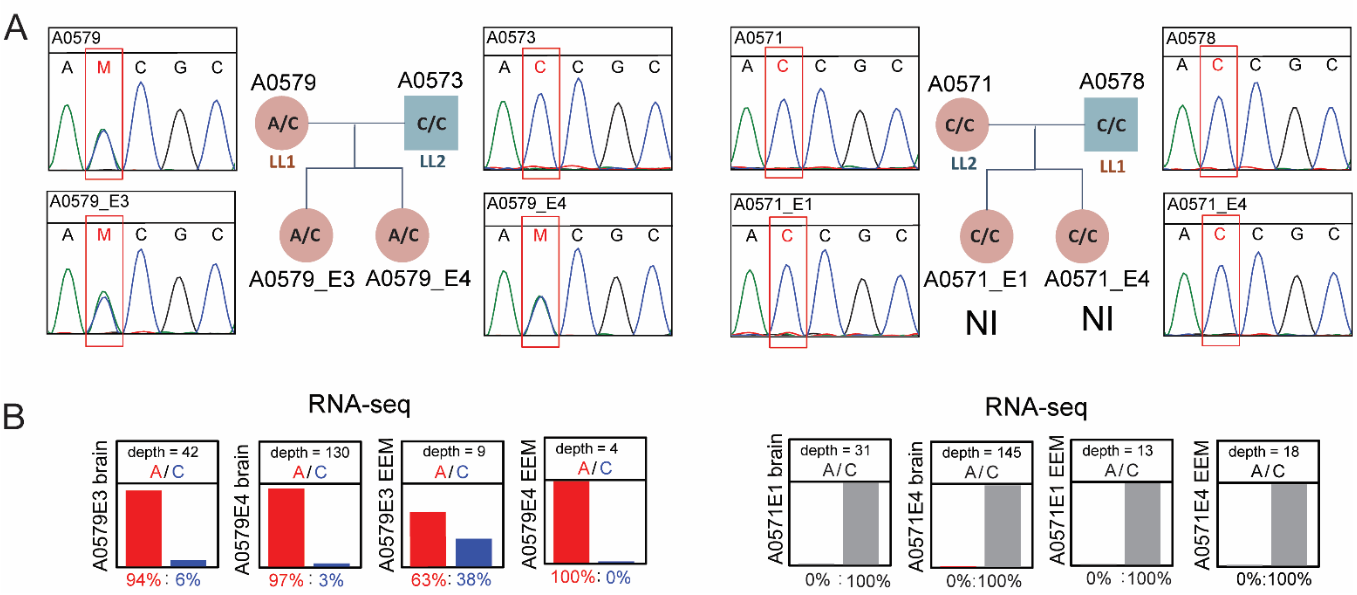

Figure S10. Programs and DNA methylation percentages at promoter CpGs quantified by PyroMark assays for four imprinted genes in the placenta.

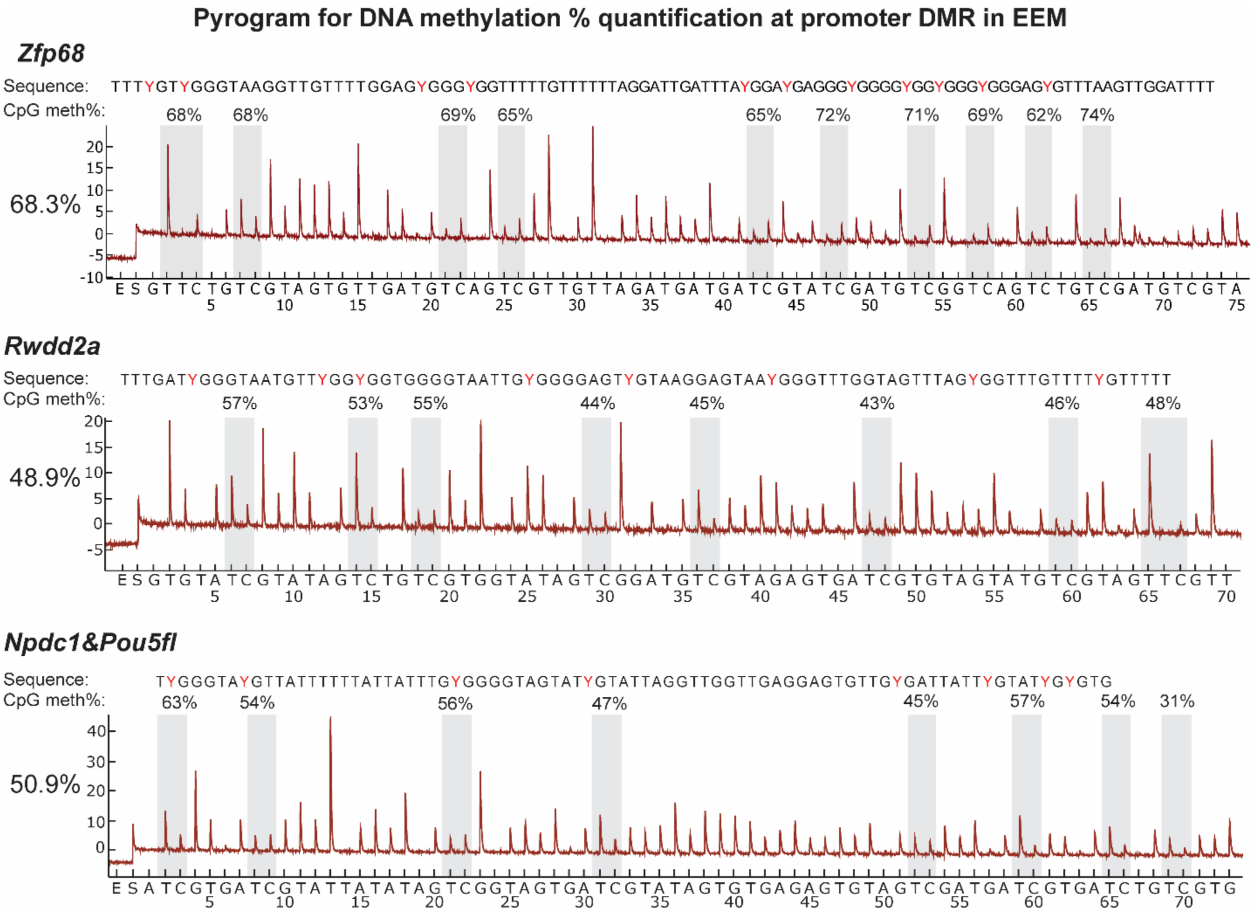

Supplement: msad022_Supplementary_Data [file msad022_supplementary_data.zip › FigureS1-S10_20221022.pdf]
