# Supplementary material for: Origin and Evolution of Marsupial-specific Imprinting Clusters Through Lineage-specific Gene Duplications and Acquisition of Promoter Differential Methylation
Source: Mol Biol Evol. 2023 Jan 31;40(2):msad022. doi: 10.1093/molbev/msad022 (PMC9937046; doi:10.1093/molbev/msad022)
Supplement: msad022_Supplementary_Data [file msad022_supplementary_data.zip › TableS1-S11_20220104.pdf]

**Table S1.** ***Monodelphis domestica*** **sample information, RNA-seq yield, and accession numbers.**

| Animal ID | Sex | Cross | Strain | Dam ID | Sire ID | Tissue Type | Age | Number of reads | Accession numbers |
|---|---|---|---|---|---|---|---|---|---|
| A0579E3 | Female | F1 | LL1XLL2 | A0579 | A0573 | embryonic head | E13.0 | 123,279,342 | GSM1099162 |
| A0579E3 | Female | F1 | LL1XLL2 | A0579 | A0573 | EEM | E13.0 | 97,126,305 | GSM1099158 |
| A0579E4 | Female | F1 | LL1XLL2 | A0579 | A0573 | embryonic brain | E13.0 | 107,001,858 | GSM1099163 |
| A0579E4 | Female | F1 | LL1XLL2 | A0579 | A0573 | EEM | E13.0 | 93,176,519 | GSM1099159 |
| A0571E1 | Female | F1 | LL2XLL1 | A0571 | A0578 | embryonic head | E13.0 | 111,441,595 | GSM1099160 |
| A0571E1 | Female | F1 | LL2XLL1 | A0571 | A0578 | EEM | E13.0 | 122,984,871 | GSM1099156 |
| A0571E4 | Female | F1 | LL2XLL1 | A0571 | A0578 | embryonic brain | E13.0 | 104,401,730 | GSM1099161 |
| A0571E4 | Female | F1 | LL2XLL1 | A0571 | A0578 | EEM | E13.0 | 102,676,227 | GSM1099157 |
| A0580E1 | Female | Parental | LL1XLL1 | A0580 | A0578 | embryonic head | E12.5 | 78,348,683 | GSM1099168 |
| A0580E1 | Female | Parental | LL1XLL1 | A0580 | A0578 | EEM | E12.5 | 89,170,950 | GSM1099164 |
| A0580E5 | Male | Parental | LL1XLL1 | A0580 | A0578 | embryonic head | E12.5 | 111,042,946 | GSM1099169 |
| A0580E5 | Male | Parental | LL1XLL1 | A0580 | A0578 | EEM | E12.5 | 80,905,285 | GSM1099165 |
| A0572E1 | Female | Parental | LL2XLL2 | A0572 | A0573 | embryonic head | E12.5 | 75,672,460 | GSM1099170 |
| A0572E1 | Female | Parental | LL2XLL2 | A0572 | A0573 | EEM | E12.5 | 57,906,830 | GSM1099166 |
| A0572E3 | Male | Parental | LL2XLL2 | A0572 | A0573 | embryonic head | E12.5 | 78,107,923 | GSM1099171 |
| A0572E3 | Male | Parental | LL2XLL2 | A0572 | A0573 | EEM | E12.5 | 76,082,440 | GSM1099167 |

The RNAseq data set was from Wang X, Douglas KC, VandeBerg JL, Clark AG, Samollow PB. 2014. Chromosome-wide profiling of X-chromosome inactivation and epigenetic states in fetal brain and placenta of the opossum, *Monodelphis domestica*. Genome Research, 24:70-83.

**Table S2. Allelic counts summary of informative SNPs in candidate imprinted genes discovered in *M. domestica* placenta.**

Allelic counts were quantified in placental RNA-seq of four F1 embryos (A0579E3, A0579E4, A0571E1, A0571E4). ref_c: reference allele counts at the SNP position; alt_c: alternative allele counts at the SNP position.

| Gene name | SNP_ID | A0579E3 | | | A0579E4 | | | A0571E1 | | | A0571E4 | | |
|---|---|---|---|---|---|---|---|---|---|---|---|---|---|
| | | ref_c | alt_c | ref% | ref_c | alt_c | ref% | ref_c | alt_c | ref% | ref_c | alt_c | ref% |
| *Pou5f3* | OMSNP0021939 | 14 | 10 | 58.3% | 10 | 8 | 55.6% | 28 | 0 | 100.0% | 21 | 6 | 77.8% |
| *Pou5f3* | OMSNP0021941 | 1 | 5 | 16.7% | 4 | 7 | 36.4% | 8 | 0 | 100.0% | 9 | 3 | 75.0% |
| *Npdc1* | OMSNP0021942 | 1 | 7 | 12.5% | 1 | 7 | 12.5% | 13 | 0 | 100.0% | 11 | 0 | 100.0% |
| *Npdc1* | OMSNP0021944 | 1 | 23 | 4.2% | 1 | 10 | 9.1% | 22 | 0 | 100.0% | 23 | 0 | 100.0% |
| *Npdc1* | OMSNP0021945 | 3 | 9 | 25.0% | 3 | 5 | 37.5% | 19 | 6 | 76.0% | 15 | 3 | 83.3% |
| *Npdc1* | OMSNP0021946 | 4 | 10 | 28.6% | 2 | 5 | 28.6% | 24 | 0 | 100.0% | 21 | 3 | 87.5% |
| *Npdc1* | OMSNP0021948 | 1 | 7 | 12.5% | 0 | 7 | 0.0% | 17 | 0 | 100.0% | 14 | 0 | 100.0% |
| *Npdc1* | OMSNP0021950 | 42 | 0 | 100.0% | 21 | 0 | 100.0% | 8 | 64 | 11.1% | 10 | 45 | 18.2% |
| *Nkrfl1* | OMSNP0127877 | 0 | 6 | 0.0% | 0 | 6 | 0.0% | 9 | 0 | 100.0% | 4 | 0 | 100.0% |
| *Nkrfl2* | OMSNP0127879 | 0 | 24 | 0.0% | 0 | 18 | 0.0% | 29 | 0 | 100.0% | 20 | 0 | 100.0% |
| *Nkrfl2* | OMSNP0127880 | 1 | 24 | 4.0% | 0 | 18 | 0.0% | 37 | 0 | 100.0% | 24 | 0 | 100.0% |
| *Nkrfl2* | OMSNP0127881 | 0 | 19 | 0.0% | 0 | 20 | 0.0% | 37 | 0 | 100.0% | 21 | 0 | 100.0% |
| *Rwdd2a* | OMSNP0052727 | 21 | 0 | 100.0% | 43 | 0 | 100.0% | 3 | 37 | 7.5% | 2 | 43 | 4.4% |
| *Rwdd2a* | OMSNP0052729 | 42 | 0 | 100.0% | 42 | 0 | 100.0% | 1 | 68 | 1.4% | 0 | 48 | 0.0% |
| *Rwdd2a* | OMSNP0052731 | 51 | 0 | 100.0% | 55 | 0 | 100.0% | 1 | 53 | 1.9% | 1 | 62 | 1.6% |
| *Zfp68* | OMSNP0060500 | 0 | 1 | 0.0% | 0 | 3 | 0.0% | 5 | 0 | 100.0% | 8 | 0 | 100.0% |
| *Ipncr5* | OMSNP0080596 | 1 | 4 | 20.0% | 0 | 3 | 0.0% | 7 | 0 | 100.0% | 13 | 0 | 100.0% |
| *Ipncr5* | OMSNP0080601 | 0 | 11 | 0.0% | 0 | 3 | 0.0% | 9 | 0 | 100.0% | 10 | 0 | 100.0% |
| *Fam169a* | OMSNP0064406 | 3 | 6 | 33.3% | 0 | 4 | 0.0% | 13 | 0 | 100.0% | 18 | 0 | 100.0% |
| *Smc6l2* | OMSNP0150599 | 5 | 0 | 100.0% | 12 | 0 | 100.0% | 0 | 4 | 0.0% | 0 | 6 | 0.0% |
| *Smc6l2* | OMSNP0150602 | 10 | 0 | 100.0% | 9 | 0 | 100.0% | 0 | 9 | 0.0% | 0 | 8 | 0.0% |
| *Smc6l2* | OMSNP0150607 | 14 | 0 | 100.0% | 20 | 0 | 100.0% | 0 | 23 | 0.0% | 0 | 7 | 0.0% |
| *Smc6l2* | OMSNP0150608 | 10 | 0 | 100.0% | 18 | 0 | 100.0% | 0 | 10 | 0.0% | 0 | 13 | 0.0% |
| *Smc6l2* | OMSNP0150609 | 20 | 0 | 100.0% | 14 | 0 | 100.0% | 0 | 26 | 0.0% | 0 | 12 | 0.0% |
| *Smc6l2* | OMSNP0150610 | 9 | 0 | 100.0% | 13 | 0 | 100.0% | 0 | 13 | 0.0% | 1 | 12 | 7.7% |
| *Smc6l2* | OMSNP0150612 | 7 | 0 | 100.0% | 15 | 0 | 100.0% | 0 | 17 | 0.0% | 0 | 9 | 0.0% |
| *Smc6l4* | OMSNP0150616 | 15 | 0 | 100.0% | 25 | 0 | 100.0% | 2 | 28 | 6.7% | 1 | 17 | 5.6% |
| *Smc6l4* | OMSNP0150620 | 23 | 0 | 100.0% | 32 | 0 | 100.0% | 0 | 24 | 0.0% | 1 | 19 | 5.0% |
| *Smc6l4* | OMSNP0150621 | 28 | 0 | 100.0% | 34 | 0 | 100.0% | 0 | 22 | 0.0% | 1 | 20 | 4.8% |
| *Ipncr4* | OMSNP0150657 | 0 | 4 | 0.0% | 0 | 3 | 0.0% | 25 | 0 | 100.0% | 5 | 0 | 100.0% |
| *Sytl5* | OMSNP0154418 | 15 | 0 | 100.0% | 12 | 0 | 100.0% | 0 | 2 | 0.0% | 0 | 6 | 0.0% |

**Table S3. Allelic counts summary of informative SNPs in candidate imprinted genes found in *M. domestica* fetal brain.**

Allelic counts were quantified in placental RNA-seq of four $F_1$ embryos (A0579E3, A0579E4, A0571E1, A0571E4). ref_c: reference allele counts at the SNP position; alt_c: alternative allele counts at the SNP position.

| Gene name | SNP_ID | A0579E3 | | | A0579E4 | | | A0571E1 | | | A0571E4 | | |
|---|---|---|---|---|---|---|---|---|---|---|---|---|---|
| | | ref_c | alt_c | ref% | ref_c | alt_c | ref% | ref_c | alt_c | ref% | ref_c | alt_c | ref% |
| *Pou5f3* | OMSNP0021939 | 4 | 102 | 3.8% | 4 | 78 | 4.9% | 88 | 0 | 100.0% | 102 | 0 | 100.0% |
| *Pou5f3* | OMSNP0021941 | 2 | 68 | 2.9% | 3 | 45 | 6.3% | 58 | 0 | 100.0% | 39 | 0 | 100.0% |
| *Npdc1* | OMSNP0021942 | 3 | 56 | 5.1% | 1 | 57 | 1.7% | 70 | 0 | 100.0% | 78 | 0 | 100.0% |
| *Npdc1* | OMSNP0021944 | 3 | 78 | 3.7% | 3 | 86 | 3.4% | 102 | 0 | 100.0% | 117 | 0 | 100.0% |
| *Npdc1* | OMSNP0021945 | 13 | 83 | 13.5% | 1 | 78 | 1.3% | 69 | 0 | 100.0% | 109 | 0 | 100.0% |
| *Npdc1* | OMSNP0021946 | 3 | 95 | 3.1% | 5 | 57 | 8.1% | 89 | 0 | 100.0% | 95 | 0 | 100.0% |
| *Npdc1* | OMSNP0021947 | 2 | 62 | 3.1% | 1 | 63 | 1.6% | 60 | 0 | 100.0% | 66 | 0 | 100.0% |
| *Npdc1* | OMSNP0021948 | 3 | 63 | 4.5% | 0 | 62 | 0.0% | 49 | 0 | 100.0% | 57 | 0 | 100.0% |
| *Npdc1* | OMSNP0021949 | 5 | 30 | 14.3% | 2 | 40 | 4.8% | 26 | 0 | 100.0% | 48 | 0 | 100.0% |
| *Npdc1* | OMSNP0021950 | 160 | 0 | 100.0% | 89 | 0 | 100.0% | 7 | 108 | 6.1% | 5 | 118 | 4.1% |
| *Nkrfl1* | OMSNP0127877 | 0 | 84 | 0.0% | 0 | 156 | 0.0% | 45 | 0 | 100.0% | 113 | 0 | 100.0% |
| *Nkrfl2* | OMSNP0127879 | 0 | 106 | 0.0% | 0 | 211 | 0.0% | 84 | 0 | 100.0% | 188 | 0 | 100.0% |
| *Nkrfl2* | OMSNP0127880 | 1 | 115 | 0.9% | 1 | 180 | 0.6% | 104 | 0 | 100.0% | 228 | 0 | 100.0% |
| *Nkrfl2* | OMSNP0127881 | 0 | 138 | 0.0% | 1 | 175 | 0.6% | 123 | 0 | 100.0% | 173 | 0 | 100.0% |
| *Rwdd2a* | OMSNP0052727 | 195 | 0 | 100.0% | 69 | 0 | 100.0% | 2 | 179 | 1.1% | 1 | 59 | 1.7% |
| *Rwdd2a* | OMSNP0052729 | 231 | 0 | 100.0% | 94 | 0 | 100.0% | 3 | 173 | 1.7% | 2 | 70 | 2.8% |
| *Rwdd2a* | OMSNP0052731 | 282 | 0 | 100.0% | 102 | 0 | 100.0% | 1 | 233 | 0.4% | 1 | 98 | 1.0% |
| *Zfp68* | OMSNP0060500 | 0 | 12 | 0.0% | 0 | 20 | 0.0% | 18 | 0 | 100.0% | 23 | 0 | 100.0% |
| *Ipncr1* | OMSNP0019332 | 123 | 12 | 91.1% | 1 | 0 | 100.0% | 0 | 198 | 0.0% | 0 | 3 | 0.0% |
| *Ipncr1* | OMSNP0019336 | 117 | 0 | 100.0% | 1 | 0 | 100.0% | 13 | 175 | 6.9% | 0 | 4 | 0.0% |
| *Ipncr1* | OMSNP0019337 | 127 | 0 | 100.0% | 2 | 0 | 100.0% | 12 | 139 | 7.9% | 0 | 6 | 0.0% |
| *Ipncr1* | OMSNP0019340 | 68 | 0 | 100.0% | 2 | 1 | 66.7% | 6 | 124 | 4.6% | 0 | 4 | 0.0% |
| *Ipncr1* | OMSNP0019341 | 105 | 0 | 100.0% | 2 | 0 | 100.0% | 14 | 170 | 7.6% | 0 | 3 | 0.0% |
| *Ipncr1* | OMSNP0019342 | 145 | 0 | 100.0% | 2 | 0 | 100.0% | 13 | 159 | 7.6% | 0 | 7 | 0.0% |
| *Ipncr1* | OMSNP0019343 | 112 | 0 | 100.0% | 1 | 0 | 100.0% | 10 | 168 | 5.6% | 0 | 2 | 0.0% |
| *Ipncr1* | OMSNP0019345 | 64 | 0 | 100.0% | 1 | 0 | 100.0% | 2 | 69 | 2.8% | 0 | 5 | 0.0% |
| *Ipncr1* | OMSNP0019353 | 35 | 0 | 100.0% | 1 | 0 | 100.0% | 5 | 75 | 6.3% | 0 | 4 | 0.0% |
| *Ipncr2* | OMSNP0127873 | 1 | 19 | 5.0% | 7 | 111 | 5.9% | 33 | 0 | 100.0% | 159 | 0 | 100.0% |
| *Ipncr2* | OMSNP0127874 | 1 | 22 | 4.3% | 4 | 107 | 3.6% | 30 | 0 | 100.0% | 174 | 0 | 100.0% |
| *Ipncr2* | OMSNP0127875 | 4 | 19 | 17.4% | 0 | 91 | 0.0% | 20 | 0 | 100.0% | 101 | 0 | 100.0% |
| *Ipncr2* | OMSNP0127876 | 2 | 34 | 5.6% | 3 | 109 | 2.7% | 31 | 0 | 100.0% | 156 | 0 | 100.0% |
| *Ipncr5* | OMSNP0080596 | 2 | 82 | 2.4% | 1 | 25 | 3.8% | 77 | 0 | 100.0% | 39 | 0 | 100.0% |
| *Ipncr5* | OMSNP0080598 | 2 | 34 | 5.6% | 3 | 15 | 16.7% | 50 | 0 | 100.0% | 27 | 0 | 100.0% |
| *Ipncr5* | OMSNP0080601 | 2 | 49 | 3.9% | 1 | 12 | 7.7% | 54 | 0 | 100.0% | 29 | 0 | 100.0% |
| *Ipncr5* | OMSNP0080603 | 43 | 0 | 100.0% | 20 | 0 | 100.0% | 0 | 38 | 0.0% | 1 | 19 | 5.0% |
| *Ipncr5* | OMSNP0080617 | 25 | 7 | 78.1% | 5 | 3 | 62.5% | 0 | 29 | 0.0% | 3 | 6 | 33.3% |
| *Fam169a* | OMSNP0064406 | 2 | 40 | 4.8% | 4 | 126 | 3.1% | 31 | 0 | 100.0% | 145 | 0 | 100.0% |
| *Fam169a* | OMSNP0064408 | 0 | 29 | 0.0% | 2 | 81 | 2.4% | 15 | 0 | 100.0% | 83 | 0 | 100.0% |

| Gene name | SNP_ID | A0579E3 | | | A0579E4 | | | A0571E1 | | | A0571E4 | | |
|---|---|---|---|---|---|---|---|---|---|---|---|---|---|
| | | **ref_c** | **alt_c** | **ref%** | **ref_c** | **alt_c** | **ref%** | **ref_c** | **alt_c** | **ref%** | **ref_c** | **alt_c** | **ref%** |
| *Smc6l2* | OMSNP0150599 | 33 | 0 | 100.0% | 90 | 0 | 100.0% | 0 | 33 | 0.0% | 0 | 74 | 0.0% |
| *Smc6l2* | OMSNP0150600 | 27 | 0 | 100.0% | 62 | 0 | 100.0% | 0 | 37 | 0.0% | 0 | 61 | 0.0% |
| *Smc6l2* | OMSNP0150601 | 15 | 0 | 100.0% | 32 | 0 | 100.0% | 0 | 6 | 0.0% | 0 | 29 | 0.0% |
| *Smc6l2* | OMSNP0150602 | 33 | 0 | 100.0% | 93 | 0 | 100.0% | 0 | 27 | 0.0% | 0 | 82 | 0.0% |
| *Smc6l2* | OMSNP0150603 | 25 | 0 | 100.0% | 45 | 0 | 100.0% | 0 | 14 | 0.0% | 0 | 51 | 0.0% |
| *Smc6l2* | OMSNP0150604 | 11 | 0 | 100.0% | 57 | 0 | 100.0% | 0 | 11 | 0.0% | 0 | 58 | 0.0% |
| *Smc6l2* | OMSNP0150605 | 14 | 0 | 100.0% | 27 | 0 | 100.0% | 0 | 5 | 0.0% | 0 | 35 | 0.0% |
| *Smc6l2* | OMSNP0150606 | 24 | 0 | 100.0% | 53 | 0 | 100.0% | 1 | 7 | 12.5% | 0 | 15 | 0.0% |
| *Smc6l2* | OMSNP0150607 | 74 | 0 | 100.0% | 229 | 0 | 100.0% | 2 | 85 | 2.3% | 1 | 208 | 0.5% |
| *Smc6l2* | OMSNP0150608 | 77 | 0 | 100.0% | 187 | 0 | 100.0% | 0 | 79 | 0.0% | 1 | 225 | 0.4% |
| *Smc6l2* | OMSNP0150609 | 88 | 0 | 100.0% | 206 | 0 | 100.0% | 0 | 81 | 0.0% | 0 | 240 | 0.0% |
| *Smc6l2* | OMSNP0150610 | 74 | 0 | 100.0% | 154 | 0 | 100.0% | 0 | 56 | 0.0% | 0 | 188 | 0.0% |
| *Smc6l2* | OMSNP0150611 | 49 | 0 | 100.0% | 114 | 0 | 100.0% | 0 | 26 | 0.0% | 0 | 106 | 0.0% |
| *Smc6l2* | OMSNP0150612 | 39 | 0 | 100.0% | 127 | 0 | 100.0% | 0 | 44 | 0.0% | 0 | 169 | 0.0% |
| *Smc6l2* | OMSNP0150613 | 30 | 0 | 100.0% | 56 | 0 | 100.0% | 0 | 12 | 0.0% | 0 | 63 | 0.0% |
| *Smc6l2* | OMSNP0150614 | 19 | 0 | 100.0% | 34 | 0 | 100.0% | 0 | 7 | 0.0% | 0 | 50 | 0.0% |
| *Smc6l4* | OMSNP0150616 | 176 | 0 | 100.0% | 421 | 0 | 100.0% | 0 | 149 | 0.0% | 1 | 438 | 0.2% |
| *Smc6l4* | OMSNP0150620 | 173 | 0 | 100.0% | 486 | 0 | 100.0% | 1 | 162 | 0.6% | 0 | 374 | 0.0% |
| *Smc6l4* | OMSNP0150621 | 282 | 0 | 100.0% | 698 | 0 | 100.0% | 2 | 286 | 0.7% | 1 | 694 | 0.1% |
| *CSNK1A1-transposed* | OMSNP0151195 | 10 | 0 | 100.0% | 31 | 0 | 100.0% | 0 | 11 | 0.0% | 0 | 33 | 0.0% |
| *Ipncr3* | OMSNP0151197 | 58 | 0 | 100.0% | 111 | 0 | 100.0% | 1 | 34 | 2.9% | 0 | 150 | 0.0% |
| *Ipncr4* | OMSNP0150657 | 0 | 34 | 0.0% | 0 | 41 | 0.0% | 28 | 0 | 100.0% | 70 | 0 | 100.0% |
| *Ipncr4* | OMSNP0150658 | 20 | 0 | 100.0% | 24 | 0 | 100.0% | 21 | 0 | 100.0% | 0 | 45 | 0.0% |
| *Sytl5* | OMSNP0154418 | 38 | 0 | 100.0% | 60 | 0 | 100.0% | 0 | 17 | 0.0% | 0 | 32 | 0.0% |

**Table S4. F1 SNP genotypes for candidate imprinted genes.**

| gene name | chr | position | A0579 _E3 | A0579 _E4 | A0571 _E1 | A0571 _E4 | A0572 _E1 | A0572 _E3 | A0580 _E1 | A0580 _E5 |
|---|---|---|---|---|---|---|---|---|---|---|
| *Pou5fl* | chr1 | 469390121 | C/T | C/T | C/T | C/T | T/T | - | T/T | T/T |
| *Npdc1* | chr1 | 469395728 | C/T | C/T | C/T | C/T | T/T | T/T | C/T | C/T |
| *Nkrfl2* | chr6 | 291750260 | C/T | C/T | C/T | C/T | C/C | C/C | C/C | T/T |
| *Rwdd2a* | chr2 | 338813088 | C/T | T/T | C/T | C/T | T/T | C/T | T/T | C/T |
| *Zfp68* | chr2 | 522422185 | C/T | C/T | C/T | C/C | C/C | C/C | C/C | C/C |
| *Ipncr1* | chr1 | 432003410 | A/C | C/C | A/C | A/C | C/C | C/C | C/C | C/C |
| *Ipncr2* | chr6 | 291639563 | A/T | A/T | A/T | A/T | - | A/A | A/T | T/T |
| *Ipncr5* | chr3 | 509558241 | A/G | A/G | A/G | G/G | A/A | A/A | G/G | G/A |
| *Fam169a* | chr3 | 49751281 | A/C | A/C | C/C | C/C | C/C | C/C | C/C | A/C |
| *Smc6l2* | chrUn | 22460755 | C/G | C/G | C/G | C/G | G/G | G/G | C/C | C/C |
| *Smc6l4* | chrUn | 22534496 | C/C | C/C | A/A | A/A | C/C | C/C | A/C | C/C |
| *Sytl5* | chrUn | 73948586 | T/T | G/T | T/T | T/T | T/T | T/T | G/T | G/G |
| *Ipncr3* | chrUn | 28488850 | A/T | A/T | T/T | T/T | A/T | A/A | A/T | A/A |
| *Ipncr4* | chrUn | 23482575 | C/T | C/T | C/C | C/C | C/T | - | C/C | - |
| *CSNK1A1-transposed* | chrUn | 28330592 | G/T | G/T | G/G | G/G | G/G | G/G | G/T | T/T |
| **Prkaa2* | chr2 | 35625423 | T/T | T/T | C/C | C/C | C/T | C/T | C/T | T/T |
| **Matn2* | chr3 | 362553338 | G/G | G/G | A/A | A/A | G/G | G/G | A/A | A/A |
| **Parp4* | chr4 | 288455210 | A/A | A/A | G/G | G/G | A/A | A/A | A/A | A/G |
| *Igf2r* | chr2 | 442547043 | A/G | A/A | A/A | A/A | A/A | A/A | G/G | A/G |

Informative SNPs are shaded in green.

## Table S5. Parental SNP genotypes for candidate imprinted genes.

| gene name | chr | position | A0579 | A0573 | A0571 | A0578 | A0572 | A0580 |
|---|---|---|---|---|---|---|---|---|
| *Pou5f3* | chr1 | 469390121 | T/T | C/T | C/T | T/T | T/T | C/T |
| *Npdc1* | chr1 | 469395728 | T/T | C/T | C/T | C/T | C/C | C/T |
| *Nkrfl2* | chr6 | 291750260 | C/T | C/C | C/C | C/T | C/C | C/T |
| *Rwdd2a* | chr2 | 338813088 | C/T | T/T | T/T | C/T | C/T | C/T |
| *Zfp68* | chr2 | 522422185 | C/C | C/T | C/T | C/C | C/C | C/C |
| *Ipncr1* | chr1 | 432003410 | C/C | A/C | A/C | C/C | C/C | C/C |
| *Ipncr2* | chr6 | 291639563 | A/T | A/A | A/A | T/T | A/A | A/T |
| *Ipncr5* | chr3 | 509558241 | G/G | A/A | A/G | A/G | A/A | G/G |
| *Fam169a* | chr3 | 49751281 | A/C | C/C | C/C | C/C | C/C | A/C |
| *Smc6l2* | chrUn | 22460755 | C/G | G/G | G/G | C/C | G/G | C/G |
| *Smc6l4* | chrUn | 22534496 | A/C | C/C | A/C | A/A | A/C | A/C |
| *Sytl5* | chrUn | 73948586 | G/G | T/T | T/T | T/T | T/T | G/G |
| *Ipncr3* | chrUn | 28488850 | A/A | C/C | C//C | C/C | A/T | A/A |
| *Ipncr4* | chrUn | 23482575 | C/T | T/T | C/T | - | C/C | C/C |
| *CSNK1A1-transposed* | chrUn | 28330592 | T/T | G/G | G/G | G/G | G/G | T/T |
| **Prkaa2* | chr2 | 35625423 | C/T | C/T | C/T | C/T | T/T | C/T |
| **Matn2* | chr3 | 362553338 | G/G | A/G | A/G | A/A | G/G | A/A |
| **Parp4* | chr4 | 288455210 | A/A | A/G | A/G | A/G | A/A | A/A |
| *Igf2r* | chr2 | 442547043 | A/G | A/A | A/A | A/G | A/A | G/G |

**Table S6. Genomic location and allelic expression profiles at informative SNP positions of six imprinted genes on unplaced scaffolds.**

| Gene Name | Type | Chr | Informative SNP ID | Informative SNP positions | Tissue | Expressed allele & percentage | | | |
|---|---|---|---|---|---|---|---|---|---|
| | | | | | | Fetal brain | | EEM | |
| *Smc6l2* | Coding | chrUn | OMSNP0150599 | 22460755 | Fetal brain&EEM | Maternal | 99.90% | Maternal | 99.80% |
| *Smc6l2* | Coding | chrUn | OMSNP0150600 | 22460828 | | | | | |
| *Smc6l2* | Coding | chrUn | OMSNP0150601 | 22460974 | | | | | |
| *Smc6l2* | Coding | chrUn | OMSNP0150602 | 22463135 | | | | | |
| *Smc6l2* | Coding | chrUn | OMSNP0150603 | 22466700 | | | | | |
| *Smc6l2* | Coding | chrUn | OMSNP0150604 | 22466752 | | | | | |
| *Smc6l2* | Coding | chrUn | OMSNP0150605 | 22467567 | | | | | |
| *Smc6l2* | Coding | chrUn | OMSNP0150606 | 22467642 | | | | | |
| *Smc6l2* | Coding | chrUn | OMSNP0150607 | 22480400 | | | | | |
| *Smc6l2* | Coding | chrUn | OMSNP0150608 | 22480958 | | | | | |
| *Smc6l2* | Coding | chrUn | OMSNP0150609 | 22482413 | | | | | |
| *Smc6l2* | Coding | chrUn | OMSNP0150610 | 22485349 | | | | | |
| *Smc6l2* | Coding | chrUn | OMSNP0150611 | 22485436 | | | | | |
| *Smc6l2* | Coding | chrUn | OMSNP0150612 | 22485907 | | | | | |
| *Smc6l2* | Coding | chrUn | OMSNP0150613 | 22489097 | | | | | |
| *Smc6l2* | Coding | chrUn | OMSNP0150614 | 22489500 | | | | | |
| *Smc6l4* | Coding | chrUn | OMSNP0150616 | 22534496 | Fetal brain&EEM | Maternal | 99.9% | Maternal | 98.30% |
| *Smc6l4* | Coding | chrUn | OMSNP0150620 | 22537177 | | | | | |
| *Smc6l4* | Coding | chrUn | OMSNP0150621 | 22538014 | | | | | |
| CSNK1A1-transposed | Coding | chrUn | OMSNP0151195 | 28330592 | Fetal brain | Maternal | 100% | - | - |
| *Ipncr3* | lncRNA | chrUn | OMSNP0151197 | 28488850 | Fetal brain | Maternal | 99.70% | - | - |
| *Ipncr4* | lncRNA | chrUn | OMSNP0150657 | 23482575 | Fetal brain&EEM | Maternal | 100% | Maternal | 100% |
| *Ipncr4* | lncRNA | chrUn | OMSNP0150658 | 23483058 | | | | | |
| *Sytl5* | lncRNA | chrUn | OMSNP0154418 | 73948586 | Fetal brain&EEM | Maternal | 100% | Maternal | 100% |

**Table S7. Primer sequences used in allele-specific pyrosequencing to validate the 8 novel imprinted genes and the known imprinted gene *Igf2r*.**

| Gene name | SNP location | Primer sequences |
|---|---|---|
| *Pou5fl* | chr1:469390121 | F: TAGATGTGGGCAGTGTAGGGGTAG<br>R: [Biotin-5]CCTATCCTGGTGACTGGTCATTG<br>Seq: GGATAGGGAAAAGGCC |
| *Ipncr1* | chr1:432003410 | F: [Biotin-5]GTTGGAAGCCAGAAGAAAGATTTA<br>R: CTCTCCCAACAATCAAAAAATAAA<br>Seq: GCAGCCCTGGGATGG |
| *Nkrfl2* | chr6:291750260 | F: GCGAAGGCCCAAGAAAGTT<br>R: [Biotin-5]AAATGCACTACTTGTGGCCTGTTC<br>Seq: GCCCAAGAAAGTTTGGT |
| *Rwdd2a* | chr2:338813088 | F: [Biotin-5]GTGCGAAACTACCTGGATCGT<br>R: CTGTCTGTCGAGGCCGTAG<br>Seq: TCAATGTCCCCCTTAG |
| *Zfp68* | chr2:522422185 | F: GGAAAAGTCCCCAGCGTAAG<br>R: [Biotin-5]CAGAGGACCCACCAAAGTACTG<br>Seq: CACCATCAGCCCCAT |
| *Npdc1* | chr1:469395728 | F: GTGACTGGATTCAGCGAAAATCAT<br>R: [Biotin-5]GTGGCACTTTGTACGTCTTAACAT<br>Seq: CAGGATTCCTAGTCAGAAG |
| *Ipncr2* | chr6:291639563 | F: TCTCTCCTGCAAGCAAGGATAAG<br>R: [Biotin-5]ACAAGACTTTCATACTGGGGTCAA<br>Seq: CTAAGGTGTTACCGGAA |
| *Ipncr5* | chr3:509558241 | F: TGCGCCGAGACATTTCCA<br>R: [Biotin-5]CCGGCTACTCCCATGGACAG<br>Seq: GGTCCGGACTCCTGG |
| *Igf2r* | chr2:442547043 | F: AGGGAGACTGTCCTGCATAGAATA<br>R: [Biotin-5]TCCGATTTCACCGACTTGGTAGTA<br>Seq: TTCCAGGCCTGGGAA |

F: PCR forward primer; R: PCR reverse primer; Seq: Pyro sequencing primer

**Table S8. Primer sequences for bisulfite sequencing and Pyromark assays to quantify DNA methylation levels at CpG islands.**

| Gene name | CGI Location | # of CpG | CGI length | Primer sequences |
|---|---|---|---|---|
| *Smc6l* | chrUn:22467679-22468575 | 74 | 897 | F: TTGTTTYGTAGATAAAGAGGG<br>R: AAATCTCCCRAACACTAACC |
| *Rwdd2a* | chr2:338812461-338812748 | 27 | 288 | F: GTAGGGGTGGTGAGAAGG<br>R: [Biotin-5]ACAACTAACCAACACCCTAAAAATAAACAC<br>Seq: GGAAGAAGTGGGGAT |
| *Npdc1* | chr1:469421970-469422201 | 18 | 232 | F: TTGGTTGGATGGTAGTTGTAT<br>R: [Biotin-5]CCCAACACCCCCCAACTTCAAC<br>Seq: AAGAGTAGAAAATAAAGGG |
| *Pou5fl* | chr1:469421971-469422201 | 18 | 231 | F:GAGTTTAAAGTTGGGTGAGTAGAGTATG<br>R: [Biotin-5]CTATCTACTACCTTTTTATACCTCCATTTCT<br>Seq: AGAGTAGAAAATAAAGGGT |
| *Nkrfl2* | chr6:291749005-291749642 | 63 | 638 | F: AGGTTTAGGGTGATTTAATAGTTT<br>R: [Biotin-5]ATTTCTACTCCAAATTAAACTCCTATA<br>Seq: GGGAGGAGGTAGGTT |
| *Zfp68* | chr2:522394632-522394856 | 16 | 225 | F: GGAGGGGAGGATTAGGGA<br>R: [Biotin-5]TCCTCTCCCAACAAACTTT<br>Seq: GGTTTTTTTTGGGGTTTTAT |

F: PCR forward primer; R: PCR reverse primer; Seq: Pyro sequencing primer

**Table S9. Genome accession numbers of the 26 vertebrate species used in the phylogenetic analysis.**

| Common name | Orders | Scientific name | Accession number |
|---|---|---|---|
| Elephant shark | Chimaeriformes | *Callorhinchus milii* | GCA_000165045.2 |
| Inshore hagfish | Myxiniformes | *Eptatretus burgeri* | GCA_900186335.2 |
| Spotted gar | Lepisosteiformes | *Lepisosteus oculatus* | GCF_000242695.1 |
| Zebrafish | Cypriniformes | *Danio rerio* | GCF_000002035.6 |
| Channel catfish | Siluriformes | *Ictalurus punctatus* | GCF_001660625.1 |
| Two-lined caecilian | Gymnophiona | *Rhinatrema bivittatum* | GCF_901001135.1 |
| Axolotl | Urodela | *Ambystoma mexicanum* | GCA_002915635.1 |
| Common toad | Anura | *Bufo bufo* | GCF_905171765.1 |
| African clawed frog | Anura | *Xenopus laevis* | GCF_001663975.1 |
| Sand lizard | Squamata | *Lacerta agilis* | GCF_009819535.1 |
| Western terrestrial garter snake | Squamata | *Thamnophis elegans* | GCF_009769535.1 |
| Leatherback sea turtle | Testudines | *Dermochelys coriacea* | GCF_009764565.2 |
| Sunda zebra finch | Passeriformes | *Taeniopygia guttata* | GCF_003957565.2 |
| South African ostrich | Struthioniformes | *Struthio camelus australis* | GCF_000698965.1 |
| Emperor penguin | Sphenisciformes | *Aptenodytes forsteri* | GCF_000699145.1 |
| Chicken | Galliformes | *Gallus gallus domesticus* | GCF_000002315.6 |
| Platypus | Monotremata | *Ornithorhynchus anatinus* | GCA_004115215.4 |
| Australian echidna | Monotremata | *Tachyglossus aculeatus* | GCA_015852505.1 |
| Koala | Diprotodontia | *Phascolarctos cinereus* | GCF_002099425.1 |
| Tasmanian devil | Dasyuromorphia | *Sarcophilus harrisii* | GCF_902635505.1 |
| Common wombat | Diprotodontia | *Vombatus ursinus* | GCF_900497805.2 |
| Gray short-tailed opossum | Didelphimorphia | *Monodelphis domestica* | GCF_000002295.2 |
| Human | Primates | *Homo sapiens* | GCA_000001405.27 |
| Dog | Carnivora | *Canis familiaris* | GCA_000002285.2 |
| Cow | Artiodactyla | *Bos taurus* | GCF_002263795.1 |
| Laboratory mouse | Rodentia | *Mus musculus* | GCA_000001635.9 |

**Table S10. Accession numbers of *Npdc1, Pou5f3, Pou5f1,* and *Tcf19* sequences used in the phylogenetic analysis.**

| Species | *Npdc1* | *Pou5f3* | *Pou5f1* | *Tcf19* |
|---|---|---|---|---|
| Spotted gar | XM_006640578.2 | XM_015366814.1 | - | - |
| Channel catfish | XM_017493244.1 | XM_017493487.1 | - | - |
| Zebrafish | NM_001128783.1 | NM_131112.1 | - | - |
| Two-lined caecilian | XM_029613509.1 | XM_029613421.1 | XM_029584871.1 | XM_029584869.1 |
| Common toad | XM_040405091.1 | XM_040404920.1<br>XM_040405516.1 | - | XM_040405920.1 |
| Axolotl | Manually annotated# | g162292.t1* | g317842.t1* | g317851.t1* |
| African clawed frog | XM_018230693.1 | NM_001087873.1<br>NM_001086363.1<br>NM_001088114.1 | - | XM_018230171.1 |
| Sand lizard | XM_033137853.1 | XM_033138020.1 | XM_033141604.1 | XM_033141603.1 |
| Western terrestrial garter snake | XM_032232444.1 | XM_032233371.1 | XM_032210810.1 | XM_032211239.1 |
| Leatherback sea turtle | XM_038375002.1 | XM_038375156.1 | XM_038371055.1 | XM_038372089.1 |
| Sunda zebra finch | XM_030286482.3 | XM_030286772.3 | - | - |
| South African ostrich | XM_009668186.1 | XM_009668185.1 | - | - |
| Emperor penguin | XM_019474955.1 | XM_009290660.1 | - | - |
| Chicken | XM_025141402.1 | NM_001309372.1 | - | - |
| Platypus | XM_029054424.1 | XM_029054193.1 | NM_001242727.1 | NM_001242725.1 |
| Australian echidna | XM_038767863.1 | XM_038767725.1 | XM_038768977.1 | XM_038768838.1 |
| Koala | XM_020981809.1 | XM_020981805.1 | XM_020974090.1 | XM_020974150.1 |
| Common wombat | XM_027849689.1 | XM_027849067.1 | XM_027876192.1 | XM_027876737.1 |
| Gray short-tailed opossum | XM_001374335.3 | XM_003339642.2 | XM_007483520.1 | XM_007483516.2 |
| Tasmanian devil | XM_012554060.2 | XM_003757510.3 | - | - |
| Human | NM_015392.4 | - | NM_002701.6 | NM_001077511.2 |
| Dog | XM_022423533.1 | - | XM_538830.3 | XM_532065.6 |
| Cow | NM_001105489.1 | - | NM_174580.3 | NM_001083684.1 |
| Laboratory mouse | XM_006497777.5 | - | NM_013633.3 | NM_001163763.1 |
| Elephant shark | - | - | XM_042346752.1 | XM_042346751.1 |
| Inshore hagfish | ENSEBUT00000004872.1 | ENSEBUT00000017993.1 | - | - |

*From AUGUSTUS *ab initio* gene predictions.
#Manually annotated sequences based on tBLASTn results.

**Table S11. Accession numbers of *Nkrf* and *Nkrfl* sequences used in the phylogenetic analysis.**

| Species | *Nkrf* | *Nkrfl1* | *Nkrfl2* |
|---|---|---|---|
| Spotted gar | XM_015351575.1 | - | - |
| Channel catfish | XM_017474170.1 | - | - |
| Zebrafish | NM_001004499.3 | - | - |
| Two-lined caecilian | XM_029607610.1 | - | - |
| Common toad | XM_040406247.1 | - | - |
| Axolotl | Manually annotated | - | - |
| African clawed frog | NM_001127862.1 | - | - |
| Sand lizard | XM_033137482.1 | - | - |
| Western terrestrial garter snake | XM_032228291.1 | - | - |
| Leatherback sea turtle | XM_038416028.1 | - | |
| Sunda zebra finch | XM_012573172.4 | - | - |
| South African ostrich | XM_009671989.1 | - | - |
| Emperor penguin | XM_019471036.1 | - | - |
| Chicken | NM_001012887.2 | - | - |
| Platypus | ENSOANT00000004133 | - | - |
| Australian echidna | XM_038748151.1 | - | - |
| Koala | XM_020975112.1 | XM_020969050.1 | g8730.t1 |
| Common wombat | XM_027861394.1 | ENSVURT00010003734.1 | XM_027861394.1 |
| Gray short-tailed opossum | XM_007507127.2 | ENSMODT00000034238.1* | ENSMODT00000034238.1# |
| Tasmanian devil | XM_012553233.2 | ENSSHAT00000045331.1 | ENSSHAT00000046927.1 |
| Human | NM_017544.3 | - | - |
| Dog | XM_005641735.3 | - | - |
| Cow | NM_001102553.1 | - | - |
| Laboratory mouse | NM_029891.3 | - | - |
| Inshore hagfish | ENSEBUT00000020355.1 | - | - |

*: ENSMODT00000034238.1 exon 1 is *Nkrfl1* based on tBLASTn analysis in opossum.
#: ENSMODT00000034238.1 exon 2 is *Nkrfl2* based on tBLASTn analysis in opossum.
